# Biased Signaling Agonists Promote Distinct Phosphorylation and Conformational States of the Dopamine D3 Receptor

**DOI:** 10.3390/ijms251910470

**Published:** 2024-09-28

**Authors:** Binod Nepal, Jessica Barnett, Frank Bearoff, Sandhya Kortagere

**Affiliations:** Department of Microbiology and Immunology, Drexel University College of Medicine, 2900 Queen Lane, Philadelphia, PA 19129, USA; nb964@drexel.edu (B.N.); jlb634@dragons.drexel.edu (J.B.); frank.bearoff@temple.edu (F.B.)

**Keywords:** β-arrestin, biased signaling, dopamine D3 agonist, dopamine D3 receptor, G-protein biased agonist, phosphoproteomics, pramipexole, SK609, unbiased agonist

## Abstract

Biased agonists of G-protein-coupled receptors (GPCRs) have emerged as promising selective modulators of signaling pathways by offering therapeutic advantages over unbiased agonists to minimize side effects. The dopamine D3 receptor (D3R), a pivotal GPCR in the central nervous system, has gained significant attention as a therapeutic target for neurological diseases, including Parkinson’s disease (PD), addiction, psychosis, depression, and anxiety. We have recently designed and tested SK609, a G-protein biased D3R selective agonist, and demonstrated its efficacy in reducing motor impairment and improving cognitive effects in a rodent model of PD. The molecular mechanism by which SK609 recruits G-protein but not β-arrestin pathways is poorly understood. Utilizing all-atom molecular dynamics simulations, we investigated the distinct conformational dynamics imparted by SK609 and the reference unbiased agonist Pramipexole (PRX). Results from these studies show that the flexibility of transmembrane 3 is key to unbiased signaling, with a ~30° and ~17° shift in tilt angle in the D3R-Gi and D3R-βarrestin2 complexes, respectively. Additionally, untargeted phosphoproteomics analysis reveals unique phosphorylation sites by SK609 and PRX in D3R. These results suggest that SK609 induces conformational changes and unique phosphorylation patterns that promote interactions with G-proteins and are not conducive for β-arrestin2 recruitment and signaling.

## 1. Introduction

G-protein-coupled receptors (GPCRs) are the largest class of cell surface receptors and bind with extracellular ligands and activate various signaling pathways across the plasma membrane [1,2,3]. GPCRs are also the largest family of proteins targeted by FDA-approved drugs [4,5,6]. GPCRs can signal through several second messenger systems, including G-proteins or β-arrestin (βarr) alone or in combination [7]. The therapeutic effects of GPCR-targeting drugs are normally achieved through the activation of distinct signaling pathways [8,9,10,11]; however, many of these compounds also activate other second messenger pathways, leading to side effects. For example, morphine, a therapeutic drug for pain management, is an unbiased μ-opioid receptor agonist. It has significant side effects, including respiratory depression, constipation, nausea, vomiting, and addiction [9]. The analgesic action of morphine is transduced by the G protein-mediated pathway, whereas adverse events are by the β-arrestin-mediated pathway [9,10,11,12,13,14,15]. The angiotensin II type 1 receptor is the target for losartan for the treatment of pulmonary arterial hypertension [16]; however, losartan treatment is associated with cardiac hypertrophy and bradycardia side effects, potentially due to activation of the β-arrestin-mediated pathway [17]. For the past decade, the concept of biased signaling that limits the activation or inhibition of a given receptor by specific ligands has been successfully explored in designing better drugs with milder side effects [9,18,19,20,21,22]. Dopamine D3 receptor (D3R) has been explored as a potential drug target for the treatment of schizophrenia, Parkinson’s disease (PD), addiction, anxiety, depression, etc. D3R is a class A GPCR and belongs to the dopamine D2-like receptor family, which couples to the inhibitory Gαi/o [23] and is distributed both in the periphery and the brain [24]. In the brain, D3Rs localize to the substantia nigra, ventral tegmental area, dorsal striatum, nucleus accumbens, islands of Calleja, olfactory bulb, prefrontal cortex, and hippocampus [25,26,27] and play a significant role in locomotion, cognition, and reward-based activities [28,29,30]. Despite the structural similarity between D3R and D2R, with an overall homology of 46% and 78% in identity in their transmembrane domains [31], D3R exhibits distinct pharmacological properties [32,33,34]. D3Rs activated by unbiased agonists such as dopamine are known to undergo long-term desensitization and pharmacological sequestration phenomena wherein the receptors change their conformation to an inactive state and localize to a hydrophobic fraction within the plasma membrane without undergoing receptor internalization [35]. While the molecular mechanisms underlying the pharmacological sequestration of D3Rs is not well understood, some studies have shown the involvement of G-protein-coupled receptor kinase (GRK) and β-arr [35,36]. In contrast, activated D2R primarily undergoes receptor internalization through a GRK- and βarr-dependent mechanism [37], while other studies have reported that D2R internalization may be independent of GRK [38]. Our previous studies using chimeric D2R/D3R segments and site-directed mutagenesis led to the identification of transmembrane 3 (TM3) and intracellular loops 2 and 3 (ICL2,3) as determinants for the desensitization of D3R [37,39,40]. Results from these studies further validate that residues Ser145/146 and Cys147 are critical for D3R sequestration and desensitization and are ligand dependent [35,39].

Over the past decades, D3R has emerged as an attractive drug target for the treatment of several neurological diseases and disorders; however, there are very few D3R selective biased agonists or antagonists. We have recently designed SK609, a G-protein biased D3R agonist, and demonstrated its efficacy in reducing L-dopa-induced dyskinesia and mild cognitive impairment in the hemiparkinsonian rodent model [41,42]. In contrast to other unbiased D3R agonists like pramipexole (PRX), SK609 does not induce D3R sequestration but promotes internalization dependent on GRK1 and clathrin/dynamin I/II, but independent of βarr1/2 and GRK-interacting protein 1 (GIT1) [43]. Additionally, SK609 promotes the short-term activation of ERK1/2, whereas PRX triggers both the short- and long-term activation of ERK1/2 [36]. These differences in signaling patterns induced by SK609 and PRX have been linked to their biased signaling properties through D3R [36]. Despite this evidence, it is unclear what conformational changes promote biased signaling in D3R. Recently, Free et al. designed and characterized a series of G-protein-biased D2R agonists that interact with the hydrophobic residues Val189 and Phe189 from TM5 and residue Ile184 of extracellular loop 2 (ECl2) [44]. The evolutionary trace-guided mutagenesis of D2R suggested that the Leu125Asn, Tyr133Leu mutants promote G-protein bias and the Ala135Arg, Met140Asp mutations lead to a βarr-biased state at D2R [45]. The residues Leu125, Tyr133, and Ala135 of D2R are also conserved in D3R and correspond to Leu121, Tyr129, and Ala131, respectively, while Met140 in D2R corresponds to Val136 in D3R. Since all these residues belong to TM3, we hypothesize that the conformation of TM3 at the cytoplasmic cleft may play a critical role in biased signaling.

Phosphorylation of the Ser/Thr/Tyr residues at the C-terminal tail and/or the ICL3 plays an important role in recruiting βarr1/2 in most typical GPCRs [46,47,48,49,50]; conversely, phosphorylation of the ICL3 has a negligible effect on βarr1/2 recruitment to D3R [35,51]. Surprisingly, the mutation of Ser145/146 in ICL2 limited the recruitment of βarr2 by D3R, activated by unbiased agonists such as dopamine and quinpirole [35]. A recent review has highlighted the molecular differences between D2R and D3R that code for their desensitization, sequestration, and endocytosis patterns and suggested additional posttranslational modifications such as glycosylation and phosphorylation to differentiate D3Rs from D2Rs [52]. Accordingly, residues Ser229 and Ser257 have been identified as sites for PKC-dependent phosphorylation at D3R [53]. Despite these molecular data, it remains unclear if biased agonism at D3Rs is dictated by differential phosphorylation, and the receptor microdomains responsible for biased agonism are unknown. We performed untargeted phosphoproteomics and all-atom molecular dynamics (MD) simulations with SK609, a G-protein-biased, and PRX, an unbiased, D3R agonist to answer these questions.

## 2. Results

### 2.1. PRX and SK609 Treatment May Promote Different Phosphorylation States of D3R

**Figure 1:**
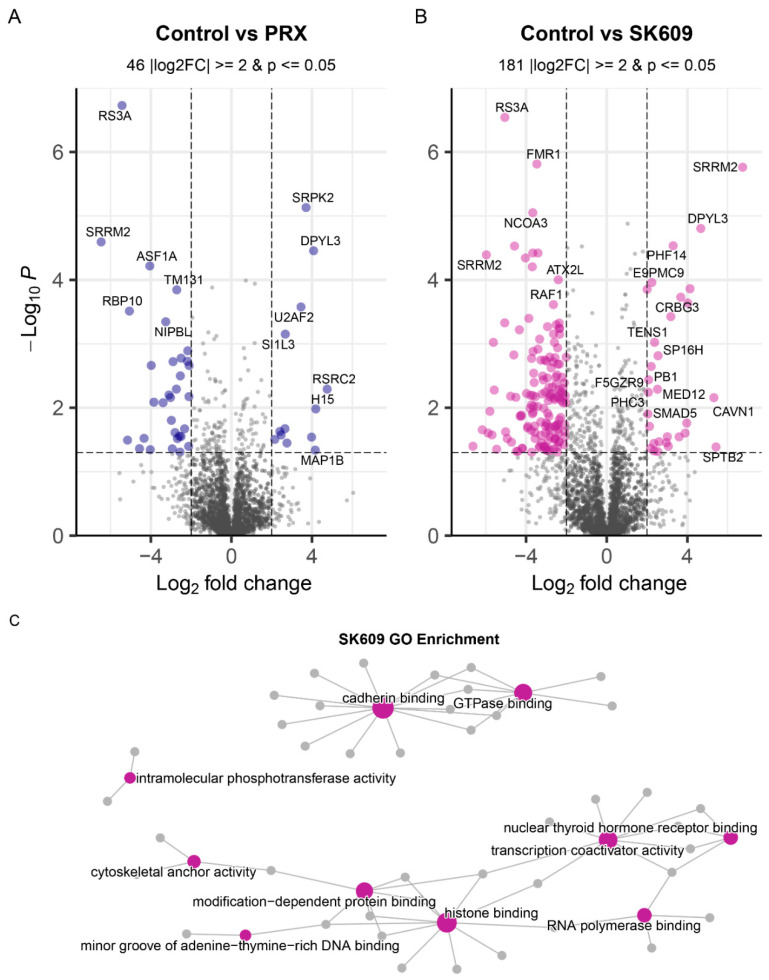
Volcano plots depicting differentially phosphorylated proteins under (**A**) PRX treatment and (**B**) SK609 treatment conditions. Proteins that are significant are marked with bigger circles and labeled, and the number of significant genes in each condition is listed above. (**C**) Functional enrichment of differentially phosphorylated proteins with SK609 treatment showed several unique molecular functions activated by G-protein recruitment Size of purple circle indicates number of proteins (grey circles) associated with the GO molecular function term.

An untargeted phosphoproteomics study was performed to identify the specific sites of phosphorylation on D3R and other proteins by SK609 and PRX treatment. Proteome-wide phosphorylation analysis of D3R-overexpressing SH-SY5Y neuroblastoma cells revealed Ser229, Ser287, and Ser295 of D3R as being phosphorylated when treated with SK609 or PRX. However, the phospho-intensities at these sites were consistently high only for SK609-treated cells, suggesting PRX-activated D3R may not prefer these sites. In addition to D3R, untargeted phosphoproteomics analysis identified 5085 phosphorus sites at a false discovery rate (FDR) of 1%, which were distributed among 2854 proteins. Pairwise comparison with a criterion of |log2FoldChange| ≥ 2 and *p*-value ≤ 0.05 indicated 46 and 181 proteins were differentially phosphorylated by PRX and SK609 treatments, respectively, compared to vehicle (Figure 1A,B). 

### 2.2. PRX and SK609 Impart Differential Pathway Utilization

Differential phosphorylation analysis was conducted on D3R-overexpressing SH-SY5Y neuroblastoma cells to determine proteome-wide changes in signaling pathway utilization by PRX and SK609 treatments (Figure S1). In total, 32 pathways were differentially phosphorylated by PRX (Table S1) and 57 by SK609 (Table S2) compared to the vehicle. The PRX and SK609 treatments shared 16 common pathways (Figure S1 insert), characterized predominantly by RNA processing. The PRX-specific pathways included functions such as RUNX1 interactions, nonsense-mediated decay, and receptor tyrosine kinase signaling (Figure S1A). Comparatively, the SK609-specific pathway list is punctuated by an abundance of terms involving RHO and RAC GTPases, suggesting a preference for G-protein-driven pathways (Figure S1B). Gene ontology (GO) functional enrichment of SK609 treated cells similarly highlights the role of GTPases (Figure 1C).

### 2.3. PRX and SK609 Promote Different Conformational Changes in D3R

**Figure 2:**
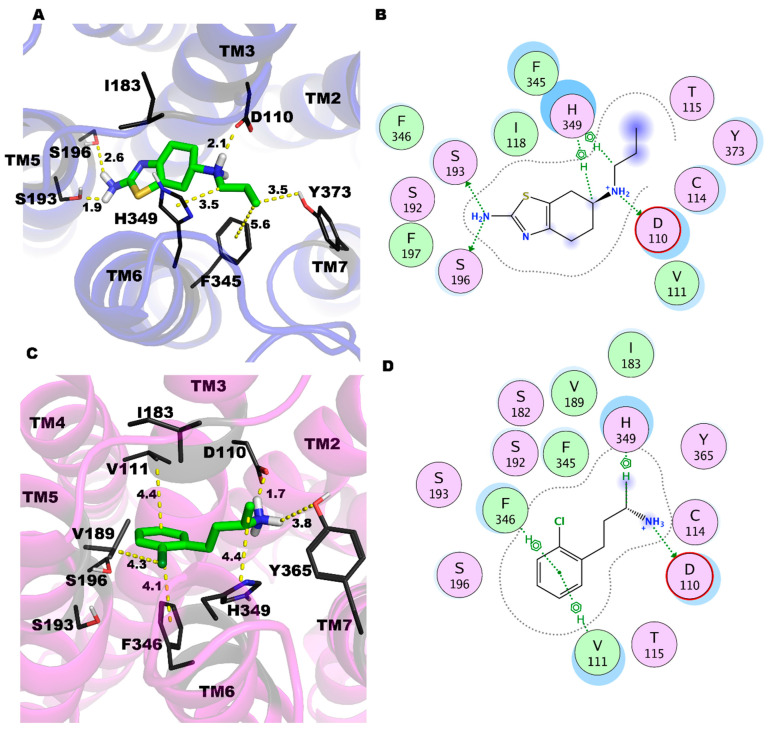
Ligand-binding pose and the ligand interaction map for the PRX (**A**,**B**) and SK609 (**C**,**D**) in D3R. D3R is shown in the cartoon model with a blue and purple color, respectively, for the D3R-PRX and D3R-SK609 complexes, respectively. PRX and SK609 are shown in the stick models with a green color. Important residues are shown as the black lines and are labeled. The helices are also labeled TM2-7. Nitrogen, oxygen, and hydrogen are shown in blue, red, and white colors, respectively. The distances are shown in the yellow dashed lines and are measured in the Å unit.

In this study, we hypothesized that PRX and SK609 induce unique conformational changes in D3R that may be responsible for differential phosphorylation states and hence the recruitment of G-proteins and/or βarr. To evaluate this, we carried out a 1 µs long MD simulation of D3R complexes with PRX and SK609 in an explicit membrane environment. The ligands were stable in the orthosteric binding pocket throughout the simulation. The binding poses and the important molecular interactions after the 1 µs simulation are presented in Figure 2, and their interaction energies with each TM domain are listed in Table 1. Both PRX and SK609 form conserved salt-bridge interactions with Asp110 and their respective protonated amine groups, which is a dominant feature of the agonist interactions of class A GPCRs. This is reflected in the highest energy change observed with TM3 movements in the simulations (Table 2). PRX makes additional H-bonds with Ser196 and Ser193 side chains (TM5) through its terminal amine group. The N-propyl group interacts with Phe346(TM6) and Tyr373(TM7) side chains. Conversely, SK609’s interactions at the orthosteric site are contributed by CH-π H-bonds from the side chains of Val111 and Phe346. The Cl atom at the ortho position of the benzene ring in SK609 increases the electron density of the ring and forms favorable aromatic interactions with Phe346 (TM6), resulting in an interaction energy ~−3.4 kcal/mol (Table 1). The Cl atom also interacts with the side chain of the residues Val189 (TM5) and His349 (TM6), which may influence its biased agonist properties. The protonated amine group of SK609 engages in H-bond interaction with the side chain of Tyr365 in TM7 with interaction energy −2.6 kcal/mol. SK609 also engages with Ile183 from ECL2, which is not observed with PRX’s interactions at the orthosteric site.

**Figure 3:**
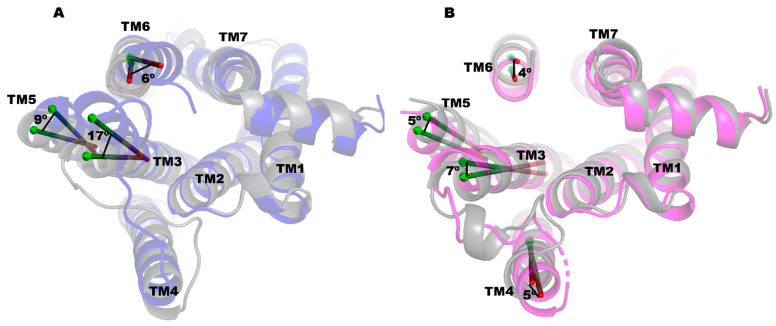
The movement of TM helices on the cytoplasmic side derived from PCA for complex (**A**) D3R-PRX in blue ribbons and (**B**) D3R-SK609 in magenta ribbons is shown with the gray helices, representing the movement from the average position. The relative displacements of the helices are measured in degrees and are shown.

**Figure 4:**
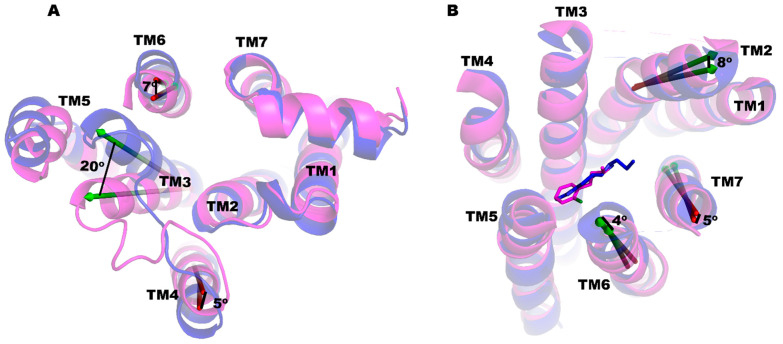
The alignment of the D3R helices in D3R-PRX (blue color) and D3R-SK609 (magenta color) complexes on (**A**) the cytoplasmic side or (**B**) extracellular side views. The PRX and SK609 are shown in the stick model in their respective cartoon colors. The relative displacements of the helices are measured in degrees and are shown.

We also performed a principal component analysis (PCA) of the trajectories from the MD simulations to analyze the conformational changes in the TM regions. The conformational dynamics at the cytoplasmic end of the TM helices are presented in Movies S1 and S2 in SI section for PRX and SK609, respectively, and the largest movement of the helices in the cytoplasmic end are depicted in Figure 3. In the PRX complex, the largest movement can be observed in the TM3 (~17°), followed by the TM5 (~9°) and TM6 (~6°). In the SK609 complex, the largest movement can be seen in the TM3 (~7°), TM5 (~6°), TM6 (~5°), and TM4 (~4°). The superposition of the TM regions in the final conformations of PRX and SK609 complexed to D3R had a RMSD of 1.46 Å (Figure 4). At the ligand binding site, TM6 tilted inward by ~4° and TM7 tilted outward by ~4° in the PRX complex relative to the SK609 complex. Additionally, TM2 tilted inward by ~8° in the PRX complex relative to the SK609 complex. The conformational changes in D3R brought by the ligands at the orthosteric binding pocket transmit toward the cytoplasmic loops, leading to the recruitment of either the G-protein, βarr, or both. At the cytoplasmic end, TM3 is tilted by ~20° toward TM6 in the D3R-PRX complex relative to D3R-SK609 complex. TM5 and TM6 in the D3R-SK609 structure are tilted inward by ~7° and ~5° relative to the D3R-PRX complex. Additionally, we also observed that the tilt angles of the TM helices were altered in these D3R ligand-bound complexes (Table 2).

Previous studies have emphasized the critical role of Ser145/146 phosphorylation in βarr recruitment, with ICL3 phosphorylation having a negligible effect [35,51]. The solvent accessible surface area of the residues Ser145/146 in the D3R-PRX complex is 93 ± 20 Å^2^ and in the D3R-SK609 complex is 71 ± 20 Å^2^. The average length of the ICL2 (the terminal distance between TM3 and TM4 on the cytoplasmic side) during the simulation was higher in the PRX complex than in the SK609 complex (21.4 ± 1.3 Å vs. 14.6 ± 0.8 Å) (Figure S2). The stretching of the ICL2 potentially helps to expose Ser145/146 residues more in the PRX complex than in the SK609 complex. Ser145/146 residues in the TM4 are in the close vicinity of the ICL2.

### 2.4. D3R-βarr2-a Complexes of PRX and SK609 Do Not Support Experimental Findings

**Figure 5:**
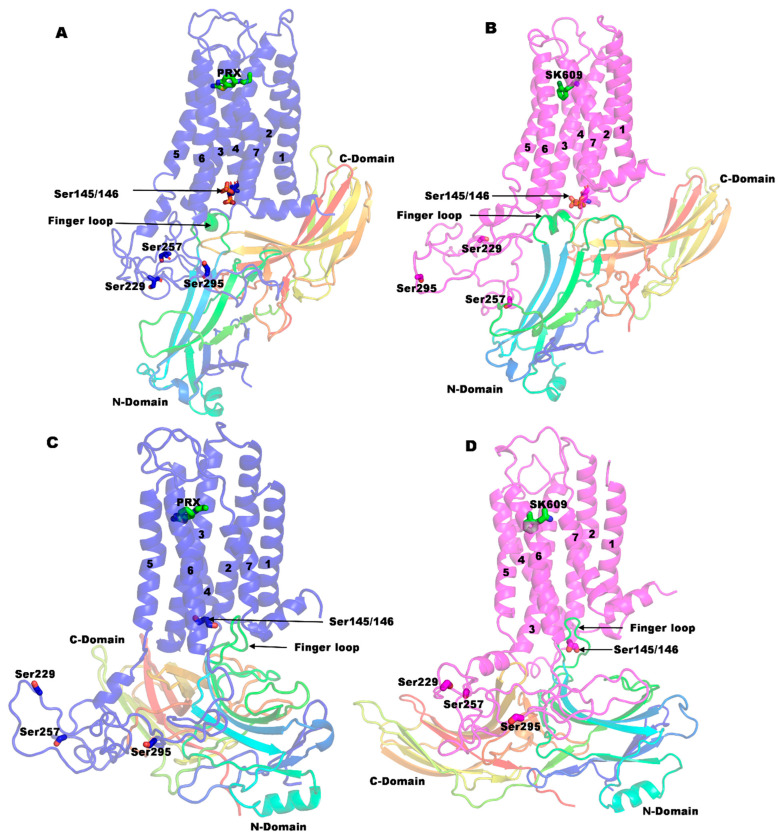
The final conformations of the D3R-βarr2 complexes, (**A**,**B**) D3R-βarr2-a complexes and (**C**,**D**) D3R-βarr2-b complexes. The blue color and magenta color represent D3R in the D3R-βarr2-a/b-PRX complexes and D3R-βarr2-a/b-SK609 complexes, respectively. βarr2 is shown in the rainbow color. The ligands are shown in the green-color stick model, and the TMs of D3R are labeled with numbers 1-7. Ser145/146/229/257/295 are shown in the stick model with nitrogen and oxygen in the blue and red color, respectively.

The equilibrated D3R-βarr2-a complexes of the PRX and SK609 from the 1 µs long MD simulation are presented in Figure 5A,B. In the D3R-βarr2-a complexes, the finger loop region does not insert deeper into the cytoplasmic cleft. In this conformation of D3R, the ICL3 is able to interact with the charged grove of the N-domain of the βarr2. The interaction energies between the various components within the complex are presented in Table 3. In the D3R-βarr2-a model, TM2, TM3, TM6, and TM7 interact with the finger loop region (residue 64–76) of βarr2 and TM3 has the largest contribution to this interaction. Similarly, ICL2 and ICL3 contribute to the βarr2 binding but ICL1 does not. The complete protein–protein interactions between D3R and βarr2 are shown in Tables S3 and S4. In the D3R-βarr2-a-PRX complex, Lys392 and Lys311 form ionic H-bonds with the Glu135 and Asp136, respectively, of βarr2. The Arg128 of the DRY motif of D3R interacts with the Asp68 of the finger loop of βarr2. Similarly, His140 and Tyr138 from the ICL1 loop of D3R make important contributions to the binding by forming H-bonds with the finger loop region (Glu67 and Asp68). In the D3R-βarr2-a-SK609 complex, Arg128 (DRY motif D3R) forms a salt bridge with Asp68 (finger loop of βarr2). Further, unlike the PRX complex, His140 and Tyr138 from the ICL1 loop of D3R do not interact with the finger loop residues Glu67 and Asp68 in the D3R-βarr2-a-SK609 complex. Experimental findings suggested that phosphorylation of Ser145 and Ser146 on D3R is critical for the recruitment of βarr2, and hence, we predict that these residues should have a significant contribution to the βarr2 binding. Interestingly, in both D3R-βarr2-a complexes, phosphorylated Ser145 and Ser146 do not contribute to βarr2 binding. Furthermore, MM-PBSA calculations (Table 4) show a positive change in interaction energy of 65.23 and 10.35 kcal/mol between the D3R and βarr2 subunits in the D3R-βarr2-a-PRX and D3R-βarr2-a-SK609 complexes, respectively, suggesting these complexes may not be favored.

### 2.5. D3R-βarr2-b Complexes Are More Plausible than the D3R-βarr2-a Complexes

The D3R-βarr2-b complexes bound to PRX and SK609 were equilibrated for a 1 µs long MD simulation (Figure 5C,D). In these models, the ICL3 loop of D3R is not proximal to interact with the positively charged grove of the βarr2 N-domain but can interact with other regions of βarr2. The interaction energies between the various components within the complexes are presented in Table 3. The interaction energies between D3R and βarr2 in D3R-βarr2-b complexes are −596.1 and −480.2 kcal/mol for the D3R-βarr2-b-PRX and D3R-βarr2-b-SK609 complex, respectively. In these models, TM2, TM3, TM6, and TM7 of D3R interact with the finger loop of βarr2 at the cytoplasmic cleft region. The largest contribution to the interaction is from TM3, followed by TM2, TM6, and TM7. Unlike in the D3R-βarr2-a complexes, all three ICLs contribute to the binding of βarr2 in the D3R-βarr2-b complexes and the contribution of the ICL1 interaction with βarr2 is significantly higher for the D3R-βarr2-b-PRX than the D3R-βarr2-b-SK609 complex (−47 vs. −8 kcal/mol, respectively). The complete set of protein–protein interactions is listed in Tables S5 and S6. Experimental evidence indicates that Ser145/146 phosphorylation of D3R is critical for recruiting βarr via H-bond interactions with the Arg66 and Lys136 of βarr2 [35]. Importantly, the Asp70 of βarr2 forms a salt bridge with the Arg128 of the DRY motif of D3R. The RMSD between the D3R-βarr2-b-PRX and D3R-βarr2-b-SK609 complexes is 2.87 Å (Figure S3). The finger loop in D3R-βarr2-b-PRX inserts ~4 Å deeper than in the D3R-βarr2-b-SK609. On the cytoplasmic side, TM2 and TM3 tilt inward by 12° and 7° and TM7 and TM6 tilt outward by 5° and 4° in the D3R-βarr2-b-PRX and D3R-βarr2-b-SK609 complexes, respectively. Interestingly, βarr2 recruitment requires the breakage of the ionic H-bonds between Asp127(TM3) and Thr64(TM2) (Figure S4 and Table 5). These H-bonds are intact in the original D3R-Gi complex (7CMU), as well as in the D3R-PRX and D3R-SK609 complexes. The interaction energies of D3R and βarr2 subunits in D3R-βarr2-b-PRX and D3R-βarr2-b-SK609 complexes are −273.93 and −198.50 kcal/mol, respectively, as determined from the MM-PBSA method. The result indicates that D3R-βarr2-b complexes are energetically more favorable than the D3R-βarr2-a complexes, and D3R-βarr2-b-PRX is more likely to exist than the D3R-βarr2-b-SK609 complex.

### 2.6. The Conformation of the Double-Mutant D3R (Leu121Asn and Tyr129Leu)-PRX Complex Is More Homologous to the D3R-SK609 Complex

**Figure 6:**
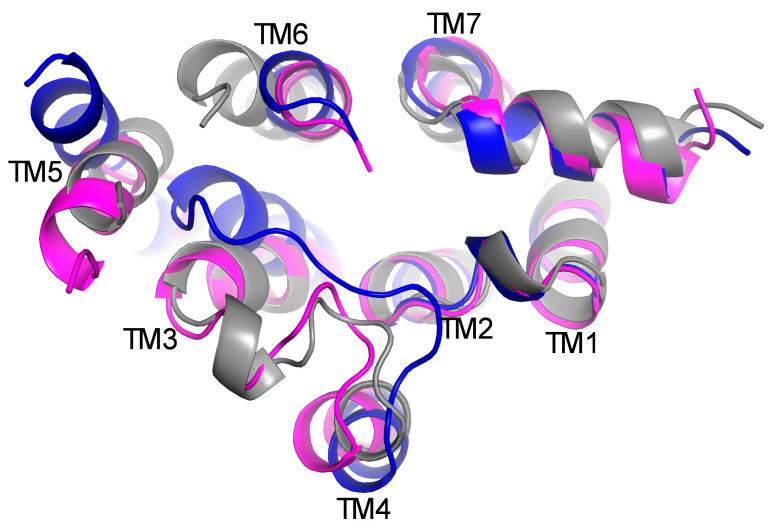
The alignment of the D3R helices in D3R-PRX (blue color) and D3R-SK609 (magenta color) with double-mutant D3R (Leu121Asn, Tyr129Leu)-PRX (gray color).

Previously, the Leu125Asn and Tyr133Leu double mutant of D2R was found to confer G-protein bias [45]. Both residues localize to TM3; however, the mechanism by which this mutant (Leu125Asn and Tyr133Leu) promotes G-protein bias is not understood. Interestingly, both residues are conserved between D3R and D2R. We hypothesize that the corresponding D3R double mutant (Leu121Asn, Tyr129Leu) would also exhibit G-protein bias, and its conformation at the cytoplasmic side cleft must be similar to that of D3R-SK609 complex. The conformational dynamics of this mutant might provide important insights regarding the G-protein-biased signaling in D3R. To understand the conformational states of the double mutants in D3R, MD simulations of the double-mutant D3R(Leu121Asn, Tyr129Leu) and in complex with PRX was performed. The average conformation of the mutant D3R(Leu121Asn, Tyr129Leu)-PRX complex was superimposed with the D3R-PRX and D3R-SK609 complexes (Figure 6). The D3R (Leu121Asn, Tyr129Leu)-PRX complex has RMSDs of 2.9 and 2.3 Å with D3R-PRX and D3R-SK609, respectively. On the cytoplasmic side, the conformation of the TM3 of the double mutant is better aligned with the D3R-SK609 complex. The local interactions of residues Leu121 and Tyr129 were analyzed (Figures S5–S7). The Leu121 side chain interacts mostly with Trp342 and Phe338. The downward movement of the Phe338 (PIF motif) and Trp442 (Toggle switch residue) side chains is required for the activation of the D3R [54]. Tyr129 lies at the interfacial region of the membrane in the TM3, and mutation Tyr129Leu reduces the tilt angle of TM3 from 30° to 24°, which mimics the conformation of TM3 in the D3R-SK609 complex. This likely may be due to Tyr129, an amphipathic residue present at the membrane interface, which may have a structural role in maintaining the tilt of TM3, which is reduced with the mutation to a hydrophobic leucine. In the D3R-PRX complex, the Tyr129 side chain interacts with Trp208 and Val207, which is not observed in the D3R-SK609 complex. In D3R (Leu121Asn, Tyr129Leu)-PRX, Leu129 side chains interact with I126 in the mutant model.

### 2.7. PRX- and SK609-Bound D3R-Gi Complexes Have Similar Conformations

**Figure 7:**
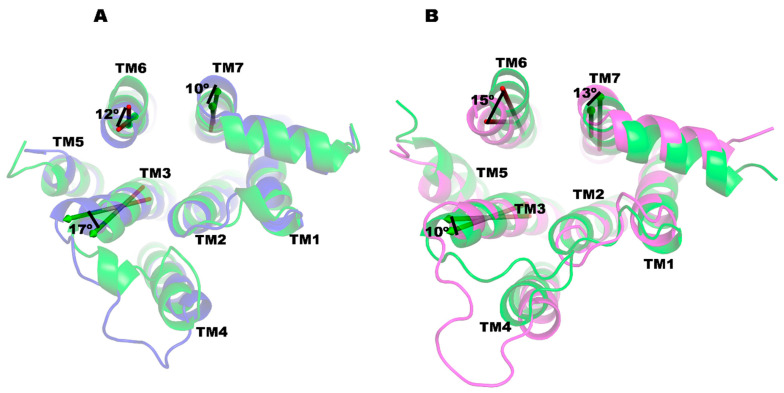
Superimposition of the D3Rs in D3R-Gi and D3R-βarr2-b complexes on the cytoplasmic side. (**A**) D3R-Gi-PRX with D3R-βarr2-b-PRX, and (**B**) D3R-Gi-SK609 with D3R-βarr2-b-SK609. The D3R in the D3R-Gi complexes is shown in green and the D3R in the D3R-βarr2-b-PRX and D3R-βarr2-b-SK609 complexes is shown in blue and magenta colors, respectively. The TM helices are labeled and the relative displacement of the angles are shown in degrees.

MD simulations were conducted with the D3R-Gi complex bound to PRX or SK609 and the superposition of D3R-Gi-PRX and D3R-Gi-SK609 complexes, showing a high level of structural similarity, with a RMSD of 2.21 Å (Figure S8A–C). The crucial protein–protein interactions between the α-subunit of the Gi protein and the D3R are listed in Tables S7 and S8 for the D3R-Gi-PRX and D3R-Gi-PRX-SK609 complexes, respectively. In both cases, the ICL3 (residues 210–321) of D3R makes several salt bridges (Arg226-Asp337, Arg222-Asp309, Arg323-Glu318, Arg305-Asp261, Arg254-Asp341, Arg323-Phe354) with the α-subunit of Gi protein. In both complexes, the α5 helix of the α-subunit of G-protein interacts with the ICL2 (residues Pro131, Val136, His137, Tyr138), TM5 (Arg219, Arg222) and TM3 (Arg128, Ala131)) of D3R. MM-PBSA calculations show that the binding enthalpies of the Gi subunit with D3R in D3R-Gi-PRX and D3R-Gi-SK609 are −287.33 and −385.08 kcal/mol, respectively, suggesting that the D3R-Gi-SK609 complex is more stable than the D3R-Gi-PRX complex. The binding enthalpies of the Gi and βarr2 subunits with D3R are comparable in the D3R-Gi-PRX and D3R-βarr2-a-PRX complexes. However, the binding enthalpy of the Gi subunit with D3R in D3R-Gi-SK609 is significantly higher (~187 kcalmol) than the binding enthalpy of the βarr2 subunit with D3R in the D3R-βarr2-a-SK609 complex. Furthermore, it was interesting to know the conformational differences in D3R in the D3R-Gi and D3R-βarr2 complexes, especially at the cytoplasmic side, which involves recruiting G-protein or βarr2. The structure of D3R in the D3R-Gi complexes was superimposed with D3R from the D3R-βarr2-b complexes (Figure 7A,B). The RMSDs between D3R-βarr2-b-PRX and D3R-Gi-PRX and between D3R-βarr2-b-SK609 and D3R-Gi-SK609 were 3.31 and 3.27 Å, respectively, for the D3R. On the cytoplasmic side of the D3R, the conformations of the TM3, TM5, and TM6 were altered in the D3R-βarr-b complexes relative to the D3R-Gi complexes. In the D3R-βarr-b-PRX complex, TM3 is tilted toward TM5 by ~17° relative to D3R-Gi-PRX. Similarly, TM6 is tilted inward by 12° and TM7 is tilted outward by 10°. The conformational differences in D3R in the D3R- βarr-b-SK609 and D3R-Gi-SK609 are also in a similar pattern as in the PRX complexes but with slightly different magnitudes of the tilt angles. The tilt angles of the TM helices with respect to the membrane normal show that the TMs, especially TM3, TM5, and TM7, in the D3R-Gi complexes are more upright than in the D3R-βarr2 complexes (Table 2).

## 3. Discussion

D3Rs, like most GPCRs, engage in G-protein-dependent and G-protein-independent signaling by recruiting βarr2. Since the discovery of biased signaling ligands and their utility in limiting the side effects of therapeutics, there has been a steady rise in the development of G-protein- or βarr-biased signaling compounds that target D2Rs [44,55,56,57]. However, the development of biased ligands at D3Rs has been very limited, with only a few rationally designed compounds [58]. D3Rs undergo long-term desensitization and pharmacological sequestration with little or no internalization when treated with unbiased ligands like dopamine, which has limited our understanding of biased signaling at D3Rs [35,52]. We have rationally designed SK609, a G-protein biased agonist of D3R with limited βarr2 recruitment that promotes complete internalization [58]. In this study, we wanted to understand the molecular mechanisms by which SK609 promotes biased agonism at D3Rs. We hypothesize that the G-protein biased agonism of SK609 in D3R results from the differential conformational dynamics of the D3R-SK609 complex relative to the unbiased D3R-PRX complex, coupled with differential phosphorylation sites at the D3R. Previous studies have highlighted the role of Ser145 and Ser146 in the recruitment of βarr2 by D3Rs activated by dopamine [35], suggesting these serine residues may be phosphorylated by GRKs. We and others have also demonstrated that Ser145, Ser146, and Cys147 are involved in agonist-induced desensitization [32,39]. Further, the Ser229 and Ser257 residues of D3R have been identified as being phosphorylated by PKC [53]. We performed untargeted phosphoproteomics on D3R-overexpressing SH-SY5Y cells treated with SK609 or PRX to understand the phosphorylation signatures at D3R and on other substrates. Results from the analysis suggested Ser229, Ser285, and Ser287 from the ICL3 loop of D3R as phosphorylation sites for SK609 but remained inconclusive for PRX. Previous studies have also corroborated that phosphorylation sites on ICL3 have a negligible effect on βarr recruitment [35,51]. Pathway analysis of differentially phosphorylated proteins suggests several G-protein-mediated pathways are phosphorylated by SK609 treatment compared to those of PRX. Previous studies on βarr recruitment by other GPCRs suggest that phosphorylation in the C-terminal tail of the GPCR and agonist-induced conformational change in the phosphorylated C-terminal tail is required to make it accessible to βarr [59]. Hence, we utilized MD simulations of SK609 and PRX in complex with D3R bound to either Gi protein or βarr2 to understand the conformational dynamics that may promote differential phosphorylation events at D3R. The higher solvent accessibility of the Ser145/146 in the D3R-PRX complex (93 ± 20 Å^2^) than in the D3R-SK609 complex (71 ± 20 Å^2^) indicates these residues may be amenable to phosphorylation by GRK. Besides the agonist-dependent phosphorylation of D3R by GRKs, Ca^2+^/calmodulin-dependent protein kinase II (CaMKII) phosphorylates Ser229 in the ICL3 region of D3R [60]. Similarly, protein kinase C (PKC)- dependent sequestration and phosphorylation of D3R at Ser229 and Ser257 have also been reported, which were independent of GRK, βarr, or caveolin 1 [53]. This suggests that SK609 may phosphorylate D3R through either CaMKII and/or PKC.

The differential phosphorylation patterns in D3R induced by SK609 and PRX are likely due to the conformational changes produced by the binding of these agonists. The results from the MD simulations suggest augmented interactions between SK609 and the residues from TM6, as well as hydrophobic interactions with residues from TM5, may play a pivotal role in the G-protein-biased signaling of SK609. Conversely, the unbiased agonist PRX exhibits robust interactions with TM5, forming two H-bonds while exhibiting weaker interactions with TM6. Notably, PRX’s H-bonds with Ser195 and Ser193 weaken the interhelical H-bond interaction between Ser196 (TM5) and Thr115 (TM3), potentially accounting for the increased flexibility of TM3 (Figure S9). In the D3R-SK609 complex, this H-bond is notably stronger. The H-bond interaction of the ligand with Ser193 in TM5 has been identified as critical for Gi/o-mediated signaling in the D2R, and the selective mutation of Ser193 to Ala has been shown to impair G-protein-mediated signaling [61]. Furthermore, studies have speculated that an agonist capable of blocking TM5 interactions while promoting ECL2 interactions would exhibit βarr bias [61]. However, this theory is contradicted by the discovery of MLS1547, a G-protein-biased D2R agonist that cannot form H-bonds with TM5 but instead interacts with the hydrophobic residues Val190 and Phe189 of TM5 [44]. This finding aligns with our results, as SK609 primarily engages with TM5 and ECL2 (Ile183) through hydrophobic interactions. Interestingly, in the κ-opioid receptor (KOR) and µ-opioid receptor (MOR), the receptor sub-domain between TM5 and ECl2 in the orthosteric binding pocket was found to be crucial for G-protein bias, while the sub-pocket in the TM2/TM3 region was found to be critical for βarr2 bias [62]. Notably, neither PRX nor SK609 interacts with TM2 in D3R. A recent study on the KOR examined the differential conformational changes imparted by G-protein-biased, βarr-biased, and balanced KOR agonists through a combination of crystal structure determination, mutagenesis, and MD simulations [63]. It was found that an agonist’s H-bond with Gln115, located in TM2, was critical for G-protein-biased signaling, while the preservation of the salt bridge between Lys227 (in TM5) and Glu297 (in TM6) was crucial for arrestin recruitment [63]. However, none of these residues are conserved in D3R, indicating that the same mechanism may not apply to D3R-biased signaling. In the angiotensin II type 1 receptor (AT1R), βarr-biased signaling was found to depend on the side chain conformation of Tyr292 in TM7 at the binding pocket [64]. The authors speculated that when Tyr292’s side chain in AT1R was oriented towards TM3, it induced a counterclockwise twist in TM7 at its proline kink, shifting the intracellular portion of TM7 towards TM3 and rotating the Arg126 of the DRY motif in a downward direction. This conformation facilitates βarr coupling but not G-protein coupling. The Tyr292 in AT1R is also conserved in D3R and corresponds to Tyr373. Surprisingly, in the D3R-Gi-PRX crystal structure, Tyr373’s side chain faces toward TM3, forming a H-bond with Asp110 [54]. This observation suggests that the mechanism proposed for AT1R βarr-biased signaling may not hold for D3R-biased signaling.

The results from the MD simulations suggest PRX and SK609 promote significantly different conformational changes in D3R. PRX binding to D3R significantly increases the tilt angle of TM3 compared to the D3R-SK609 complex. A comparison of the tilt angles in the D3R-βarr2-b complexes and the D3R-Gi complex indicates that βarr2 recruitment necessitates more extensive tilting of TM3, TM5, and TM7 than Gi protein recruitment. Consequently, TM3’s flexibility is essential for unbiased signaling in D3R. Principal component analysis (PCA) of the MD trajectories further supports this observation, demonstrating that TM3 exhibits high flexibility in the PRX complex compared to the SK609 complex, favoring unbiased signaling. This finding is corroborated by the results from the G-protein-biased double-mutant D3R(Leu121Asn, Tyr129Leu)-PRX, whose conformation is structurally more homologous to the D3R-SK609 complex. The movement of the TM7 toward TM3, which decreases the distance between the TM3 and TM7 at the cytoplasmic cleft region, has been proposed to promote G-protein-biased signaling in MOR [65]. In agreement with this hypothesis, the distance between TM3 and TM7 was increased by ~2 Å in the D3R-βarr2-b complexes compared to in the D3R-Gi complexes (Table 6) indicating that flexibility in TM3 would be favorable for unbiased signaling. Interestingly, the analysis of the protein–protein contacts between the GPCR and Gαi on several GPCR-G protein complexes shows that the major contributors to the interactions are TM3(23.1%), ICL2, TM7, H8 (15.4% each) and ICL1, TM2, TM5, and TM6 (7.7% each), which highlights the prominent role of TM3 [66].

Due to the absence of a D3R-βarr1/2 crystal structure, the actual conformation of the D3R-βarr complex remains unknown. To address this, we designed two models of the D3R-βarr2 complexes, named D3R-βarr2-a and D3R-βarr2-b, derived from known crystal structures of Neurotensin-βarr1 and β1-Adrenergic-βarr1 complexes, respectively. Yin et al. proposed that GPCRs with a long ICL3 tend to adopt conformations akin to the D3R-βarr2-a model, predicated on the interaction of phosphorylated long ICL3 with the positively charged groove of βarr [67]. However, in D3R, ICL3 phosphorylation plays a negligible role in βarr recruitment [51], and the phosphorylated Ser145/146 in D3R does not interact with βarr2 in this model. Energetics and available experimental findings suggest that D3R-βarr2-b conformation is a more plausible representation of the D3R-βarr2 complex. In this model, phosphorylated Ser145/146 significantly contributes to βarr2 binding by forming salt bridges with the Arg66 and Lys136 of βarr2, aligning with the experimental observations [35].

## 4. Methods

### 4.1. Untargeted Phosphoproteomics

Human neuroblastoma cells (SH-SY5Y cells) were grown to confluence in 6-well plates and were treated with a vehicle, SK609 (1 μM) or PRX (10 nM), for 5 min at 37 °C. Cells were then collected in 100 µL of lysis buffer (50 mM Tris/1 mM EDTA + protease + phosphatase inhibitor+ PMSF) and whole-cell lysates were stored at −80 °C. Samples in triplicate were subject to an untargeted phosphoproteomics study using standard protocols at the Weill Cornell Medical Center genomics and proteomics core facility. Briefly, the proteins were precipitated with acetone and in-solution tryptic digestion was performed, followed by phosphopeptide enrichment. The phosphopeptides were subject to LC-MS/MS for analysis. MS data were searched against the Uniprot human protein database, with a parameter-setting phosphorylation as a dynamic modification. For relative quantitation, the phosphopeptide intensities were log transformed and normalized using the median intensity value in each sample. One-way ANOVA was used to compare the three groups and multiple hypothesis correction was performed by the Benjamani–Hochberg method [68]. Pairwise comparisons were performed using Tukey HSD [69]. Data analysis and plotting were carried out in R.

### 4.2. Pathway and GO Enrichment Analysis

The phosphorylation intensity values of the PRX- and SK609-treated groups were compared to the control using the pairwise Tukey HSD test, and differentially phosphorylated proteins were defined as *p* < 0.05. The lists of differentially phosphorylated proteins were converted to the ENSEMBL gene namespace before submission for GO enrichment and pathway analysis using the g:Profiler GOst [70], KEGG [71], Reactome [72], and WikiPathways [73] data sources.

### 4.3. Molecular Modeling and MD Simulations

The crystal structure of D3R (7CMU [54]) was obtained from the RSCB protein data bank [74]. The D3R protein structure was prepared for MD simulations by modeling the missing ICL3 loop with MODELER software version 10.4 [75]. SK609 and PRX were built using the BUILDER module of MOE and minimized, and the protonated forms [76] were docked at the orthosteric binding site with GOLD software version 2022.3 [77]; minimization was carried out with MOE software version 2022 [78] using an AMBER force field [79]. The docked protein complexes were inserted into a membrane patch (dimensions are provided in Table 7) composed of POPC (palmitoyl-oleoyl-phosphatidylcholine) 75%, Cholesterol 12.5%, and POPI24 (palmitoyl-oleoyl-phosphatidylinositol-(4,5)-bisphosphate with protonation on P4) 12.5%. The protein was neutralized with 0.1M KCl solution. Water molecules were represented by the TIP3 model [80]. The force field for the ligands was generated using CHARMM General Force Field (CGenFF) software (version 2.5.1) [81]. The list of the systems generated for the MD simulations is provided in Table 7. Each system listed in Table 7 was initially equilibrated in six different steps for MD using NAMD software version 2.14 [82], with a CHARMM36m force field [83,84] for protein and lipids. Initially, the protein backbone atoms, lipid head groups, ligand atoms, and water molecules and ions were restrained with 0.1 kcal harmonic potential. The restraints were gradually released in five different steps. Finally, the system was freely equilibrated for 5 ns with 2 fs time steps. The production run was carried out on an Anton2 supercomputer [85] for 1–2 µs simulation time with 2 fs time steps. Simulations were run on an isobaric–isothermal ensemble (NPT), with the ensemble mode at 310 K and 1 bar, using a Nose–Hoover thermostat [86] and the Martyna, Tobias, and Klein (MTK) barostat [87]. The cutoff distances for nonbonded interactions were determined automatically by Anton2 [85]. The coordinates were saved every 1 ns of the simulation for the analysis. The RMSD plots of all MD trajectories are provided in Figure S10.

### 4.4. Modeling of the PRX- and SK609-Bound D3R-βarr2 Complexes

MD simulation of the D3R-βarr2 complexes in the presence of PRX and SK609 was carried out to understand the conformational states of D3R. No crystal structure of the βarr1/2 complex with D3R or with any other dopamine receptors has been determined to date. One of the important interactions in the formation of the GPCR-βarr complex is the insertion of the finger loop domain of βarr into the cleft within the cytoplasmic side of the GPCRs [67,88,89,90,91,92,93]. An evaluation of the available crystal structures of various class A GPCR-βarr complexes suggested that the relative orientation of the long axis of the βarr is largely varied [67,94]. For example, the alignment of the β1-adrenoceptor (β1AR)-βarr1 complex (PDB ID: 6TKO) and neurotensin receptor1 (NTR1)-βarr1 complex (PDB ID: 6PWC) shows the angle between the long axis of βarr1 in those complexes is ~90° [67]. Further, the long axis of the βarr1 in complex with β1AR remains almost parallel to the membrane plane, whereas a ~20° tilt is observed in the NTR1-βarr1 complex. βarr1 and βarr2 share a high level of sequence identity (75%) and structural similarity (Figure S11) despite some functional differences in terms of desensitization, endocytosis, and signaling. In the finger loop region (60–76 βarr1, 61–77 βarr2), both share 94% sequence identity and 100% sequence similarity. Since the crystal structure of the full-length βarr2 with any GPCR is not available except for the phosphorylated C-terminal peptides derived from some GPCRs [95,96], the structure of the βarr1-GPCR complexes described above serves as a good template to model βarr2-GPCR complexes. In this study, two models of D3R-βarr2 complexes based on the NTR1-βarr1 complex (PDB ID 6PWC) and β1AR-βarr1 complex (PDB ID: 6TKO) were modeled and are referred to as D3R-βarr2-a and D3R-βarr2-b complexes, respectively. The generated models were simulated in an explicit membrane environment and in complex with SK609 or PRX, equilibrated until the root mean squared deviations (RMSDs) were stable, and continued to for 1µs of production run. In both models, D3R was phosphorylated at Ser145/146.

### 4.5. Molecular Mechanics/Poisson–Boltzmann Surface Area (MM/PBSA) Calculations

Protein–protein and protein–ligand binding energies of the D3R-G-protein and D3R-βarr2 complexes were estimated by the MM/PBSA calculation. The calculations were carried out with the GROMACS gmx-MMPBSA program [97]. Charmm36m force fields were converted into the GROMACS force field format using the CHRAMM-GUI server [98]. The trajectories were converted into GROMACS format using the MDtraj program [99]. The MM/PBSA calculations were performed in a membrane environment (memopt = 1), with a hydrophobic thickness (mthick) of 3.8 nm, a dielectric constant of the membrane set at (emem) 7.0, and an internal dielectric constant (indi) of 20.0.

## 5. Conclusions

In conclusion, we have shown that the differential signaling behavior of SK609 and PRX in D3R is caused by their distinct conformational dynamics. PRX induces a larger dynamic movement and tilting of TM3 compared to SK609, which is required for βarr2 recruitment. The recruitment of βarr2 necessitates a wider cleft formed by TM3, TM5, TM6, and TM7 on the cytoplasmic side of D3R than what is needed for G-protein recruitment. In addition to these differential conformational dynamics, the untargeted phosphoproteomics analysis revealed that SK609 and PRX impart unique phosphorylation sites. Ser229, Ser287, and Ser295 were more distinctly phosphorylated with SK609 than with PRX. We also proposed the possibility that Arg66 and Lys136 in βarr2 undergo salt-bridge interactions with Arg128 and Glu324 in D3R. We suggest that mutations of Arg128 and Glu324 in D3R would inhibit βarr2 recruitment. Additionally, our future studies are aimed at examining the effect of mutations to residues of Ser229, Ser287, and Ser295 in D3R and their role in G-protein-biased signaling by SK609.

## Figures and Tables

**Table 1:** The average interaction energies of SK609 and PRX with different TM helices, TM3-TM7 and Ile183, in the ECL2 of D3R in various complexes. The calculations are presented from the last 500 ns of 1–2 µs simulation.

Complex	TM3	TM5	TM6	TM7	ILE183
D3R-PRX	−55.78 ± 5.5	−6.8 ± 1.8	4.3 ± 18.6	2.5 ± 12.3	2.0 ± 9.3
D3R-PRX-Gi	−45.31 ± 9.8	−6.4 ± 2.7	−1.6 ± 24.9	5.5 ± 8.0	0.3 ± 3.6
D3R-βarr2-a-SK609	−48.1 ± 8.8	−6.4 ± 2.2	−0.8 ± 8.9	2.3 ± 10.1	3.1 ± 3.1
D3R-βarr2-b-PRX	−29.6 ± 17.0	−10.1 ± 2.5	−9.3 ± 14.9	1.2 ± 0.5	2.2 ± 6.0
D3R- SK609	−66.2 ± 6.3	−5.0 ± 1.1	−3.4 ± 5.1	−1.2 ± 2.5	−0.3 ± 1.6
D3R- SK609-Gi	−66.7 ± 7.6	−5.3 ± 1.1	−7.2 ± 10.7	0.6 ± 2.2	−0.4 ± 3.3
D3R-βarr2-a-SK609	−68.6 ± 4.1	−5.5 ± 0.8	−1.7 ± 5.2	−1.4 ± 1.4	−0.2 ± 1.5
D3R-βarr2-b-SK609	−65.7 ± 8.3	−5.7 ± 1.7	−4.6 ± 4.7	−0.3 ± 2.5	−0.5 ± 1.8

**Table 2:** The average tilt angle in degrees between the TM axis and the bilayer normal from the last 500 ns of 1–2 µs of the MD simulation.

Complex	TM1	TM2	TM3	TM4	TM5	TM6	TM7
D3R-PRX	27 ± 3	29 ± 3	30 ± 3	6 ± 3	34 ± 3	7 ± 3	18 ± 4
D3R-SK609	22 ± 3	27 ± 3	25 ± 3	7 ± 3	32 ± 3	12 ± 4	23 ± 4
D3R(Leu121Asn, Tyr129Leu)-PRX	25 ± 3	29 ± 3	24 ± 3	6 ± 3	32 ± 3	17 ± 5	20 ± 4
D3R-βarr2-b-PRX	32 ± 3	32 ± 2	30 ± 2	13 ± 3	28 ± 3	7 ± 3	25 ± 4
D3R-βarr2-b-SK609	29 ± 3	30 ± 2	30 ± 2	7 ± 2	31 ± 3	8 ± 3	31 ± 3
D3R-Gi-PRX	32 ± 5	24 ± 3	17 ± 3	17 ± 3	12 ± 4	7 ± 3	11 ± 4
D3R-Gi-SK609	35 ± 4	26 ± 3	19 ± 2	13 ± 4	21 ± 4	11 ± 3	10 ± 7

**Table 3:** Average interaction energies between the different subunits in D3R-βarr2 complexes. The energies are in the kcal/mol. “fl” indicates finger loop region (residue 64–76) of βarr2. Calculations are presented from the last 500 ns of the 1 µs simulation.

Ligand	D3R_βarr2	TM2_fl	TM3_fl	TM6_fl	TM7_fl	ICL1_ βarr2	ICL2_ βarr2	ICL3_ βarr2
D3R-βarr2-a complexes								
PRX	−296.8 ± 51.1	−5.4 ± 6.2	−24.5 ± 10.5	−20.4 ± 7.3	−1.4 ± 1.31	1.87 ± 11.6	−50.7 ± 11.6	−163.1 ± 42.2
SK609	−364.2 ± 41.2	−0.2 ± 0.6	−78.2 ± 6.5	−9.3 ± 5.8	−0.3 ± 0.7	12.9 ± 10.3	−89.7 ± 12.4	−146.7 ± 29.7
D3R-βarr2-b complexes								
PRX	−596.1 ± 70.1	−54.2 ± 7.8	−67.4 ± 10.4	−22.6 ± 5.3	−9.3 ± 2.5	−46.8 ± 14.0	−97.6 ± 12.7	−247.4 ± 55.0
SK609	−480.2 ± 52.1	−17.9 ± 6.2	−63.5 ± 2.9	−17.6 ± 3.2	−6.2 ± 1.8	−8.1 ± 9.6	−113.6 ± 19.4	−214.4 ± 43.1

**Table 4:** The binding enthalpies in kcal/mol of the protein–ligand and protein–protein interactions in various complexes determined from the MM-PBSA method calculated from the last 500 ns of the MD trajectories. The protein–ligand interaction indicates the interaction of the D3R unit either with PRX or SK609. The protein–protein interaction indicates the interaction of the D3R either with a Gi or βarr2 subunit.

Complex	Protein–Ligand	Protein–Protein
D3R-PRX	−25.03 ± 2.63	
D3R-SK609	−41.65 ± 4.60	
D3R-βarr2-a-PRX	−28.88 ± 4.06	65.23 ± 18.77
D3R-βarr2-a-SK609	−40.86 ± 3.68	10.35 ± 24.38
D3R-βarr2-b-PRX	−21.15 ± 3.91	−273.93 ± 27.52
D3R-βarr2-b-SK609	−38.90 ± 3.55	−198.50 ± 18.95
D3R-Gi-PRX	−20.36 ± 15.11	−287.33 ± 24.29
D3R-Gi-SK609	−36.17 ± 2.70	−385.08 ± 35.78

**Table 5:** Average H-bond distances in Å of some important interactions within the D3R in different complexes. Calculations are presented from the last 500 ns of the simulation for the βarr2 complexes and last 1000 ns for the G-protein complexes.

Complex	D187_H354	D127_T63	D127_T64
D3R-PRX	7.2 ± 1.7	4.9 ± 1.6	1.9 ± 0.8
D3R-SK609	10.3 ± 2.0	1.8 ± 0.2	1.9 ± 0.7
D3R(Leu121Asn, Tyr129Leu)-PRX	10.3 ± 1.7	1.8 ± 0.3	1.7 ± 0.2
D3R-βarr2-b-PRX	13.9 ± 0.7	2.1 ± 1.0	5.8 ± 1.2
D3R-βarr2-b-SK609	14.6 ± 1.3	8.9 ± 0.6	6.3 ± 0.8
D3R-Gi-PRX	9.3 ± 1.6	1.8 ± 0.3	2.4 ± 1.2
D3R-Gi-SK609	9.1 ± 1.8	3.0 ± 1.5	2.2 ± 1.0

**Table 6:** The average distances (in Å) between TM3 and TM7, and between TM2 and TM6 at the cytoplasmic cleft region of the various D3R complexes. Calculations are presented from the last 500 ns of the simulation for the βarr2 complexes and last 1000 ns for the G-protein complexes.

Complex	TM3-TM7	TM2-TM6
D3R-PRX	18.7 ± 0.9	17.5 ± 0.9
D3R-SK609	23.6 ± 1.0	16.7 ± 0.8
D3R(Leu121Asn, Tyr129Leu)-PRX	22.9 ± 0.9	19.7 ± 0.8
D3R-βarr2-b-PRX	27.1 ± 0.6	18.6 ± 0.7
D3R-βarr2-b-SK609	27.1 ± 0.6	19.4 ± 0.6
D3R-PRX-Gi	25.7 ± 1.0	18.8 ± 0.5
D3R-SK609-Gi	23.7 ± 1.1	20.4 ± 0.5

**Table 7:** The list of systems considered for MD simulations.

Systems	Number of Lipids	Patch Dimension Å × Å (Å^2^)	Simulation Time
1. D3R-PRX	131	73 × 73	1 µs
2. D3R-SK609	131	73 × 73	1 µs
3. D3R(Leu121Asn, Tyr129Leu)-PRX	133	73 × 73	1 µs
4. D3R-βarr2-a-PRX	327	105 × 105	1 µs
5. D3R-βarr2-a-SK609	331	105 × 105	1 µs
6. D3R-βarr2-b-PRX	323	103 × 103	1 µs
7. D3R-βarr2-b-SK609	333	103 × 103	1 µs
8. D3R-Gi-PRX	301	101 × 101	2 µs
9. D3R-Gi-SK609	301	101 × 101	2 µs

## Data Availability

The original contributions presented in the study are included in the article/supplementary material, further inquiries can be directed to the corresponding author/s.

## Supplementary Materials

The following supporting information can be downloaded at https://www.mdpi.com/article/10.3390/ijms251910470/s1.
