# Supplementary material for: Biased Signaling Agonists Promote Distinct Phosphorylation and Conformational States of the Dopamine D3 Receptor
Source: Int J Mol Sci. 2024 Sep 28;25(19):10470. doi: 10.3390/ijms251910470 (PMC11476979; doi:10.3390/ijms251910470)
Supplement: Supplementary file 1 [file ijms-25-10470-s001.zip › ijms-3196314 SM.pdf]

Supporting Information

Biased signaling agonists promote distinct phosphorylation and conformational states of the Dopamine D3 receptor

Binod Nepal[+], Jessica Barnet[+], Frank Bearoff[+,#] and Sandhya Kortagere[+*]

[+]Department of Microbiology and Immunology, Drexel University College of Medicine, Philadelphia, PA, 19129, USA.

[#]Current address: Department of Neurology, Temple University Lewis Katz School of Medicine, Philadelphia, PA, 19140, USA.

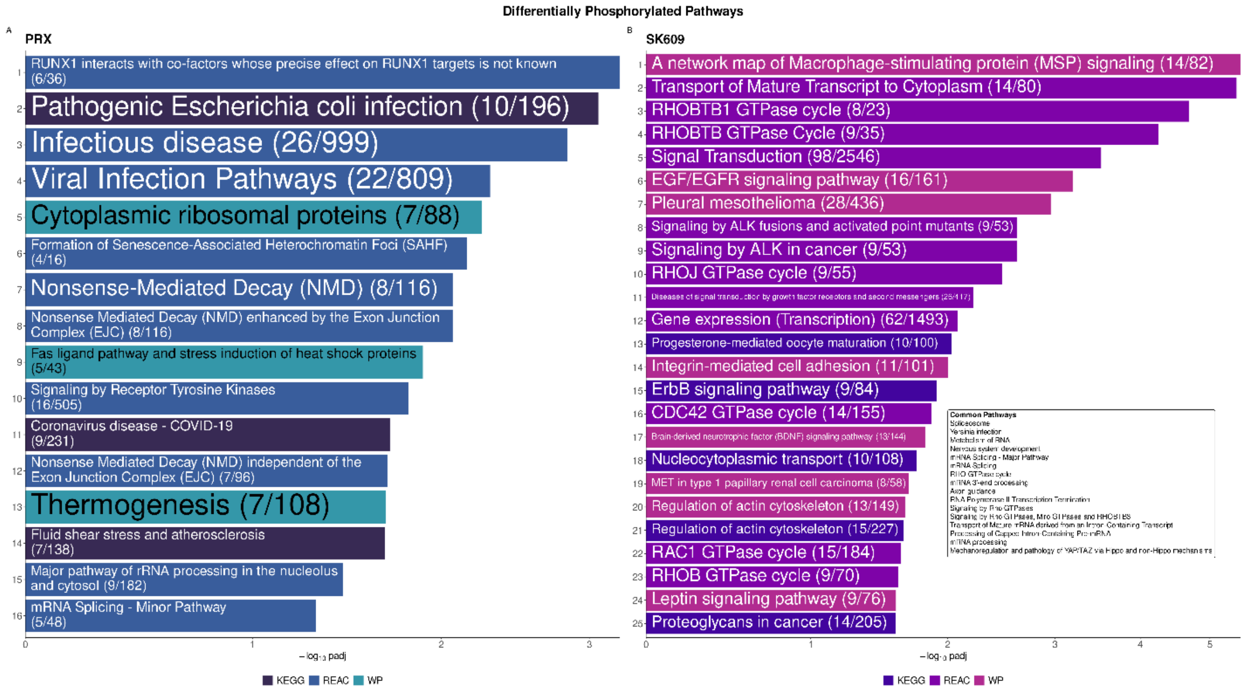

Fig S1. Functional enrichment pathway analysis on the list of differentially phosphorylated proteins induced by PRX- (a, left) and SK609-treated cells (b, right) was performed. Enriched pathways common to both treatments are listed in the inset. Pathway sources included KEGG, Reactome, and WikiPathways as indicated by color legend. Pathways are ordered by significance in descending order. The fraction of differentially phosphorylated proteins found in each pathway is indicated in parenthesis.

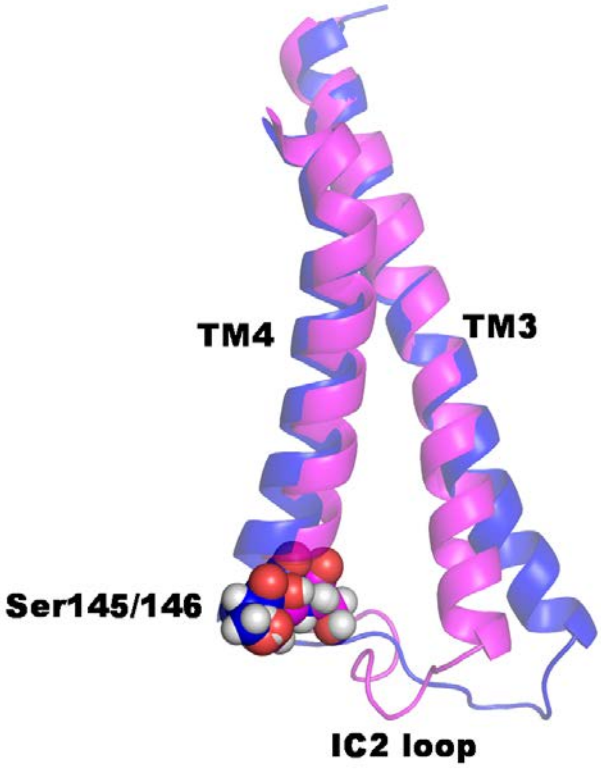

Fig S2. The conformations of the TM3 and TM4 in D3R-PRX(Blue) and D3R-SK609(Magenta) complexes. The residues Ser145/146 are shown in the sphere model with nitrogen in blue, oxygen in red and hydrogen in white colors.

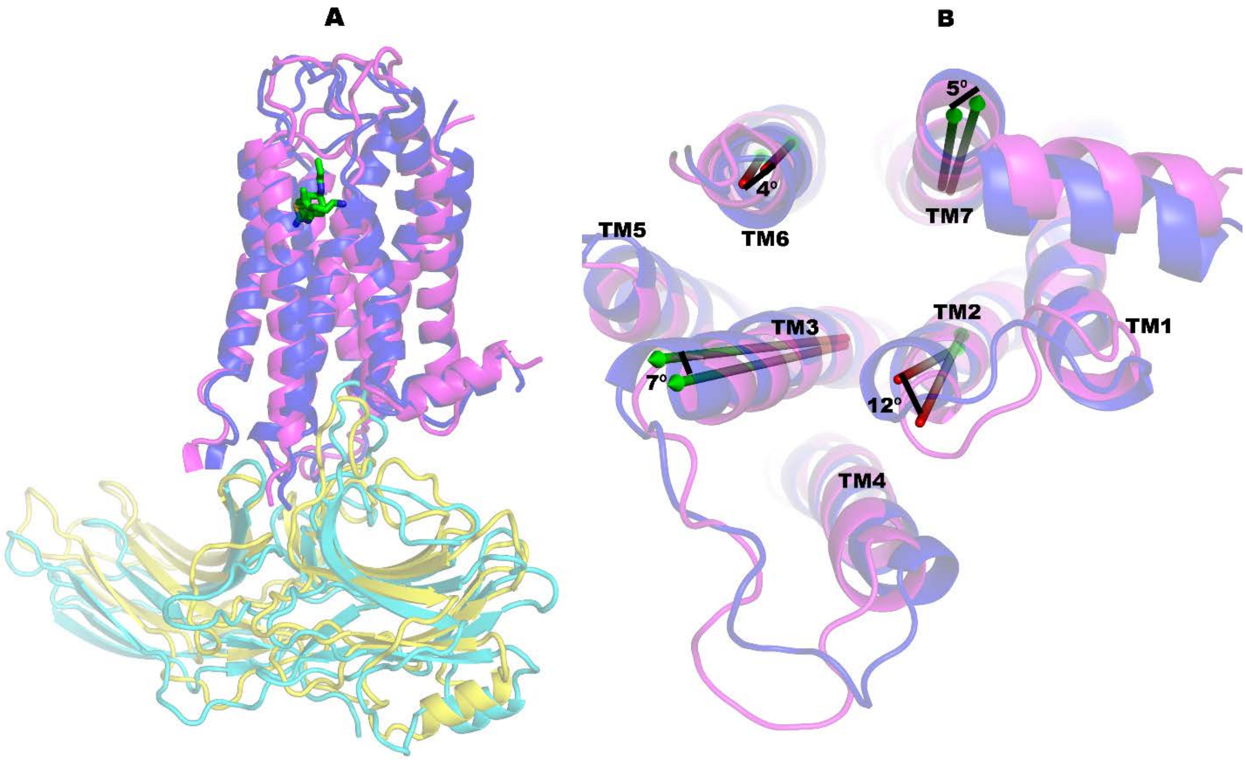

Fig S3. A) The superimposition of the D3R-βarr-b-PRX(blue and cyan color) and D3R-βarr-b-SK609 (magenta and yellow color) complexes. **B)** The cytoplasmic side view. The TM helices are labeled with numbers. The displacement of the TM helices in PRX and SK609 are measured in the angles (degree) and are shown.

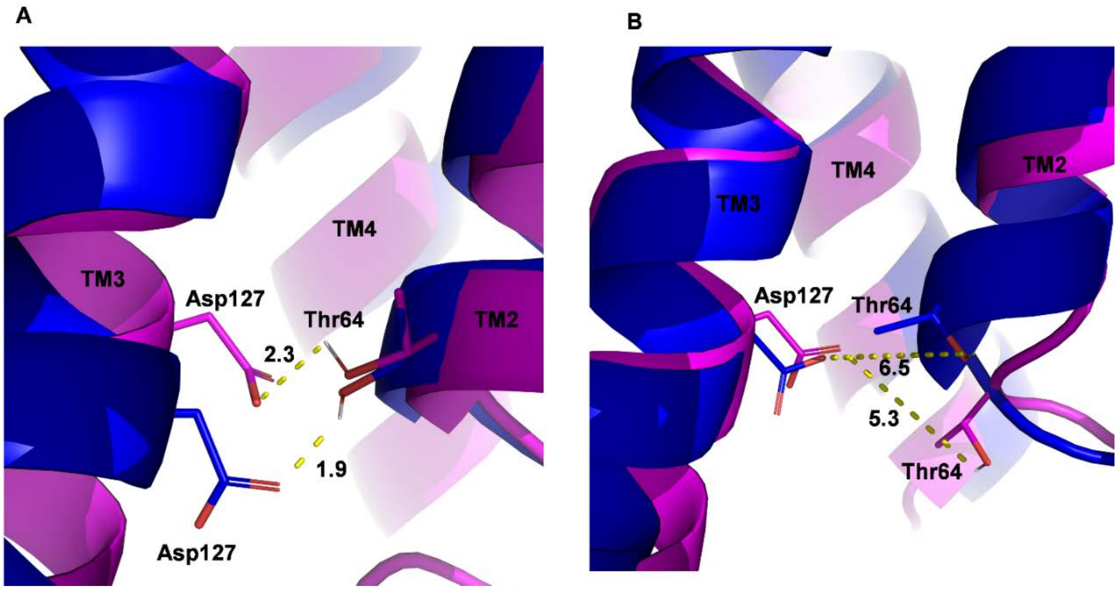

Fig S4. H-bond interaction between Asp127(TM3) and Thr64 (TM2). **A)** D3R-PRX (blue color) and D3R-SK609(magenta color), **B)** D3R-βarr2-b-PRX (blue color) and D3R-βarr2-b-SK609(magenta color). Side chains of residues Asp127 and Thr64 are shown in the stick models.

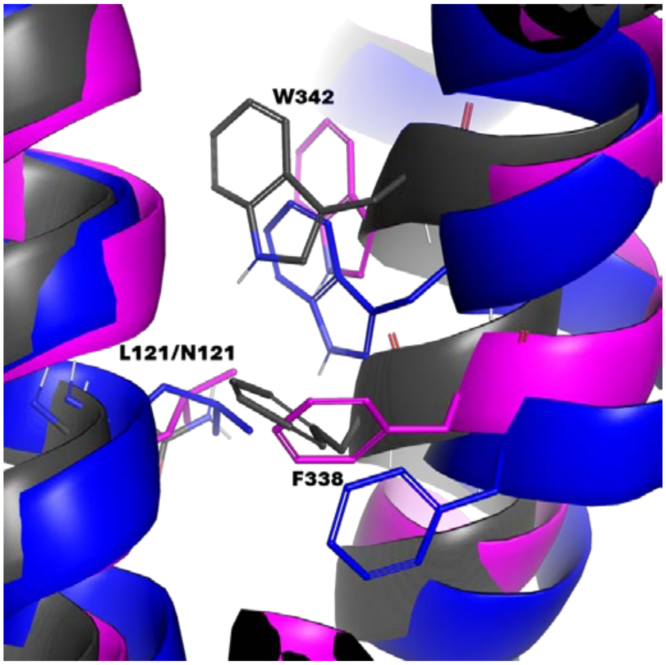

Fig S5. Conformation and the orientation of the side chains near residue 121 in D3R-PRX(blue color), D3R-SK609(magenta color) and mutant D3R (Leu121Asn, Tyr129Leu)-PRX(gray color) complexes. Side chains of residues Leu121, Phe338 and Trp342 are shown in the stick models.

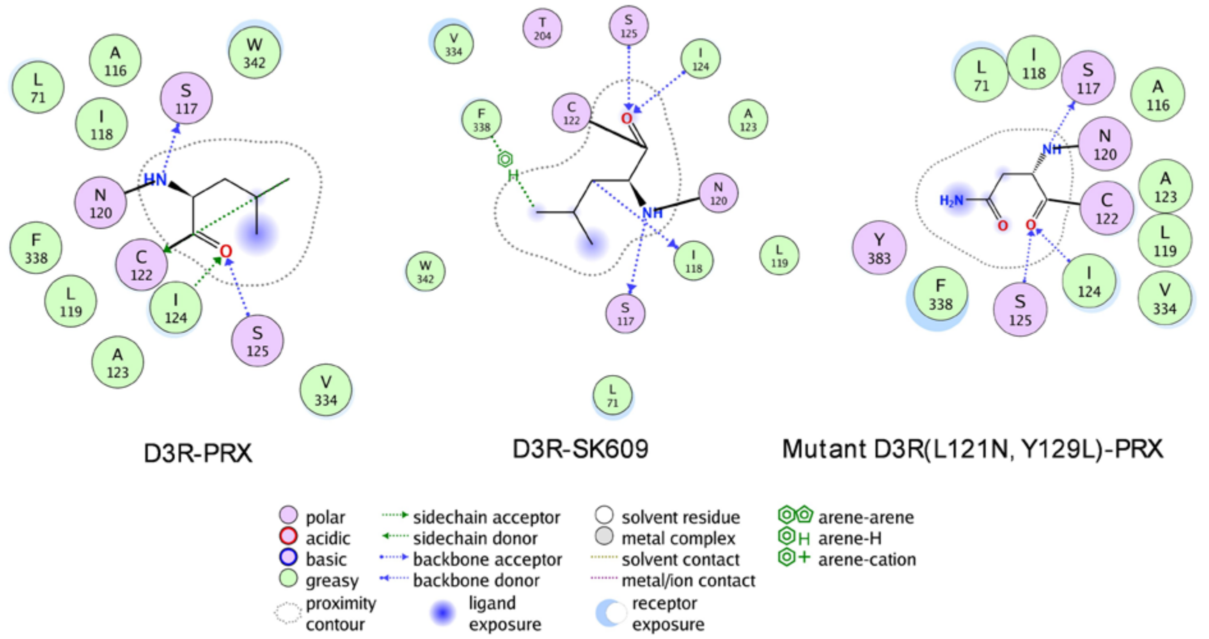

Fig S6. Side chain interaction map for the residue 121 in D3R-PRX, D3R-SK609 and mutant D3R(Leu121Asn, Tyr129Leu)-PRX complexes.

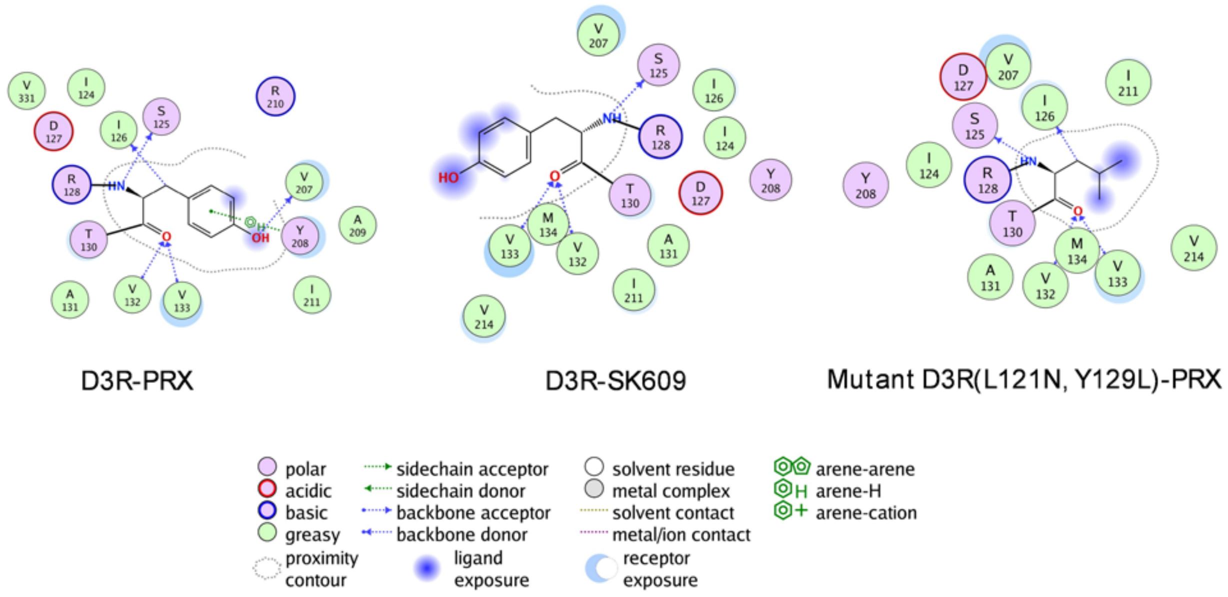

Fig S7. Side chain interaction map for the residue 129 in D3R-PRX, D3R-SK609 and mutant D3R(Leu121Asn, Tyr129Leu)-PRX complexes.

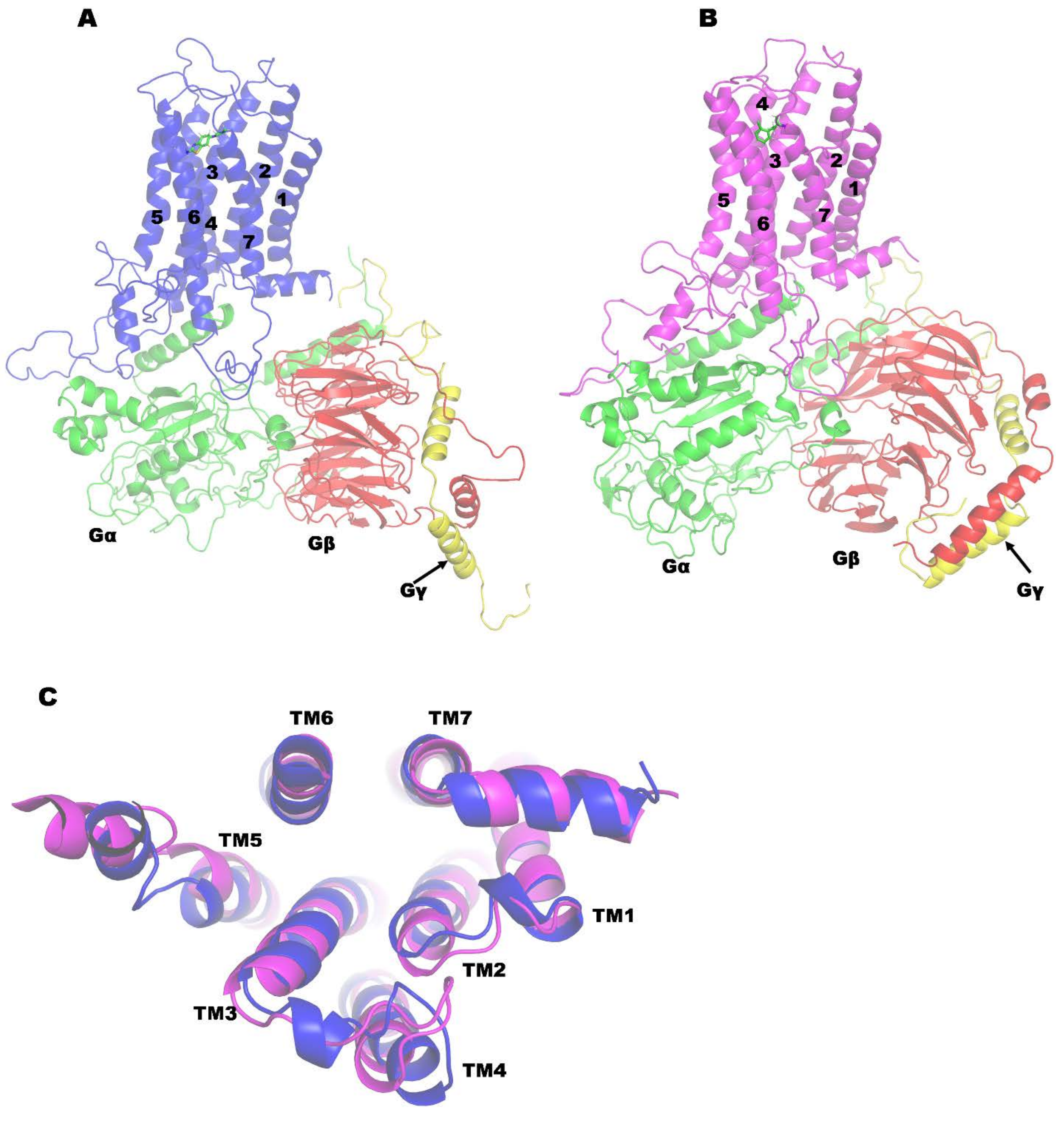

Fig S8. The final conformations of the **A)** D3R-Gi-PRX and **B)** D3R-Gi-SK609 complexes of 2μs MD simulation. **C)** The superimposed image of the two complexes at the cytoplasmic side. The D3R is colored blue in D3R-Gi-PRX and colored magenta in D3R-Gi-SK609 complexes. The TM helices are labeled with numbers.

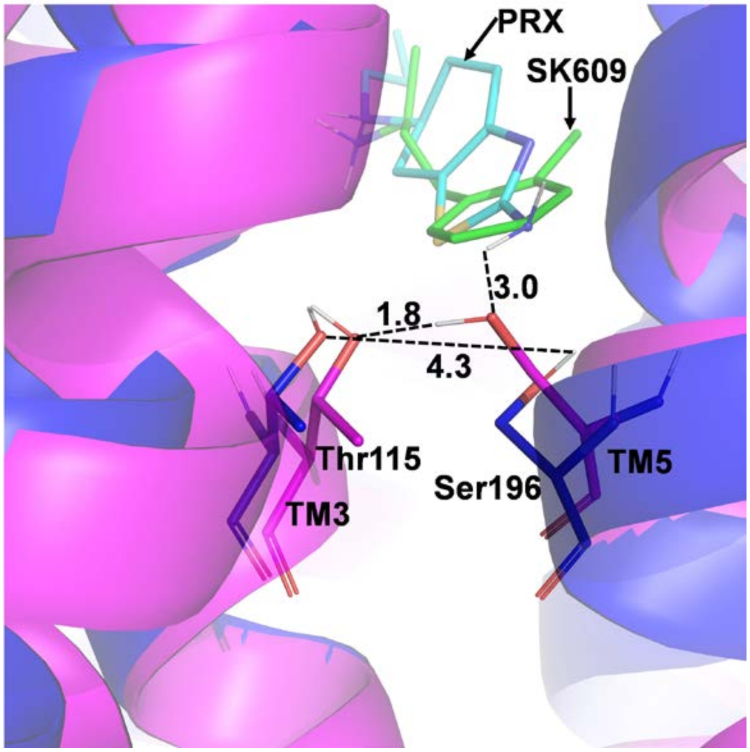

Fig S9. Side chain H-bond interaction between Thr115 and Ser196. The residues are shown in the stick model. The blue color represents the D3R-PRX complex and magenta color represents D3R-SK609 complex. The H-bond distances shown are in the Å unit.

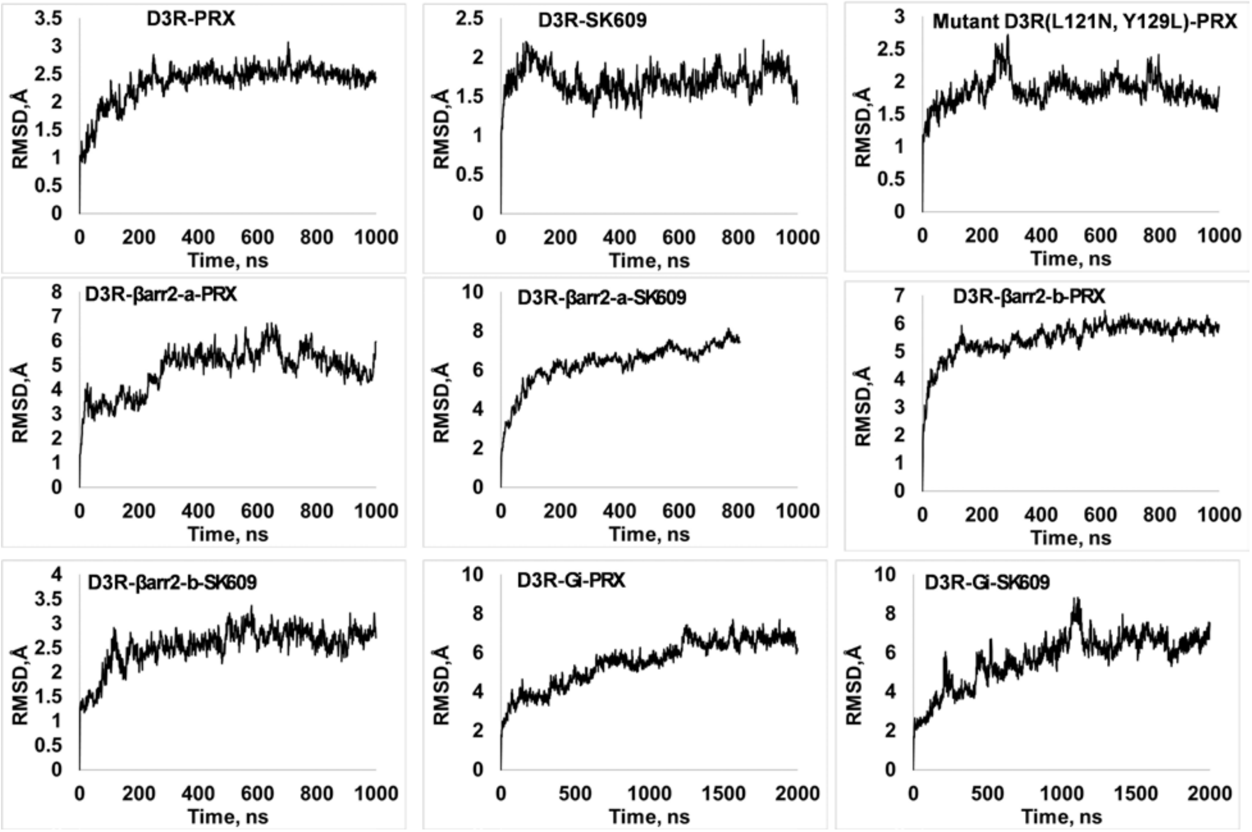

Fig S10. RMSD plots of the MD trajectories of various SK609 and PRX complexes.

A

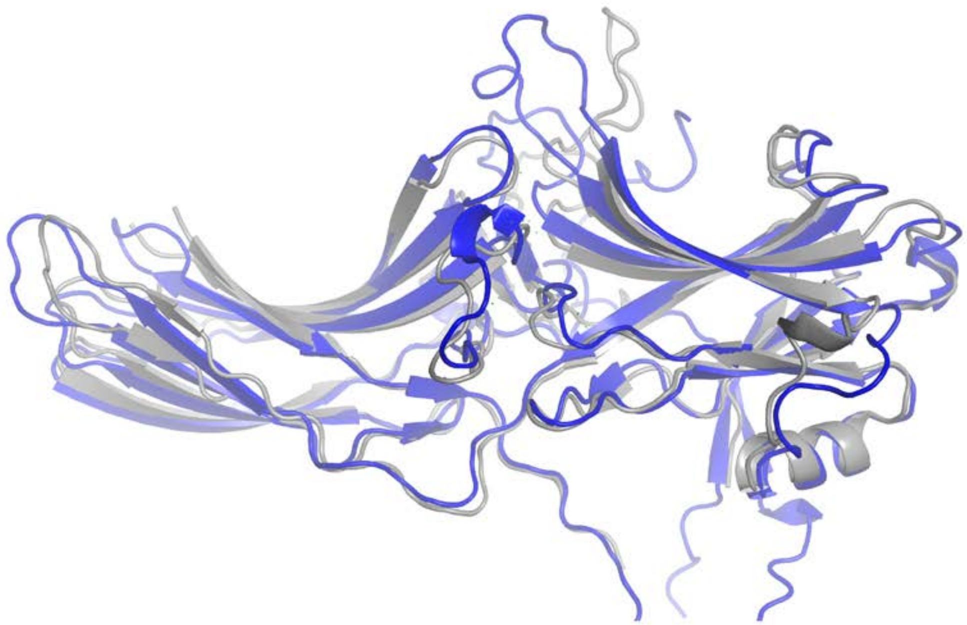

B

```
ARRB2_HUMAN      MGEKPGTRVFKKSSPNCKLTVYLGKRDFVDHLDKVDPVDGVVLVDPDYLKDRKVFVTLTC 60
ARRB1_HUMAN      -MGDKGTRVFKKASPNGKLTVYLGKRDFVDHIDLVDPVDGVVLVDPEYLKERRVYVTLTC 59
                    . *******:*** **************:* ************:***:*:*:*****

ARRB2_HUMAN      AFRYGREDLDVLGLSFRKDLFIATYQAFPPVPNPPRPPTRLQDRLLRKLGQHAHPFFFTI 120
ARRB1_HUMAN      AFRYGREDLDVLGLTFRKDLFVANVQSFPPAPEDKKPLTRLQERLIKKLGEHAYPFTFEI 119
                 **************:******:*. *:***.*:  :* ****:**::***:**:** * *

ARRB2_HUMAN      PQNLPCSVTLQPGPEDTGKACGVDFEIRAFCAKSLEEKSHKRNSVRLVIRKVQFAPEKPG 180
ARRB1_HUMAN      PPNLPCSVTLQPGPEDTGKACGVDYEVKAFCAENLEEKIHKRNSVRLVIRKVQYAPERPG 179
                 * **********************:*::****:.**** **************:***:**

ARRB2_HUMAN      PQPSAETTRHFLMSDRSLHLEASLDKELYYHGEPLNVNVHVTNNSTKTVKKIKVSVRQYA 240
ARRB1_HUMAN      PQPTAETTRQFLMSDKPLHLEASLDKEIYYHGEPISVNVHVTNNTNKTVKKIKISVRQYA 239
                 ***:*****:*****: **********:******:.********:.*******:******

ARRB2_HUMAN      DICLFSTAQYKCPVAQLEQDDQVSPSSTFCKVYTITPLLSDNREKRGLALDGKLKHEDTN 300
ARRB1_HUMAN      DICLFNTAQYKCPVAMEEADDTVAPSSTFCKVYTLTPFLANNREKRGLALDGKLKHEDTN 299
                 *****.*********  * ** *:**********:**:*::*******************

ARRB2_HUMAN      LASSTIVKEGANKEVLGILVSYRVKVKLVVSRGG--------DVSVELPFVLMHPKPHDH 352
ARRB1_HUMAN      LASSTLLREGANREILGIIVSYKVKVKLVVSRGGLLGDLASSDVAVELPFTLMHPKPKEE 359
                 *****:::****:*:***:***:***********        **:*****.******::.

ARRB2_HUMAN      IPLPRPQSAAPETDVPVDTNLIEFDTNYATDDDIVFEDFARLRLKGMKDDDYDDQLC--- 409
ARRB1_HUMAN      PP----HREVPENETPVDTNLIELDTN---DDDIVFEDFARQRLKGMKDDKEEEEDGTGS 412
                  *    :  .**.:.********:***   *********** ********. :::

ARRB2_HUMAN      ------         409
ARRB1_HUMAN      PQLNNR         418
```

Fig S11. **A)** Superposition of the human βarr1(PDB ID 6KTO, gray color) and βarr2 (alpha-fold2 generated, blue color). **B)** The sequence alignment of the human βarr1 and human βarr2.

Table S1-2. Pathways that differentially phosphorylated by PRX (**Table S1**) and by SK609 (Table-S2) are attached as excel files due to the large size.

Table S3. Protein-protein contacts between the D3R and βarr2 in the D3R-βarr2-a-PRX complex. **DIH**, **DH** and **D** denote ionic H-bond, H-bond and van der Waals interaction respectively.

Type	D3R	βarr2	Energy, Kcal/mol	Dist, Å
DIH	Lys392	Glu135	-26.15	3.57
DIH	Lys311	Glu135	-24.10	3.91
DH	Lys311	Asp136	-5.63	3.77
DH	Ser233	Leu80	-3.99	3.87
DH	HSE140	Glu67	-3.67	3.73
DH	Tyr138	Asp68	-3.43	3.70
D	Pro267	Phe245	-3.38	4.09
DIH	Arg128	Asp68	-3.28	3.90
D	Leu312	Thr137	-3.26	4.06
DH	Ser233	Lys78	-2.90	3.44
D	Arg128	Leu72	-2.87	3.84
D	Val234	Leu316	-2.46	4.21
D	Pro236	Leu316	-2.25	3.75
D	Leu215	Leu74	-2.15	4.08
D	Val136	Phe245	-1.85	3.94
D	Met330	Val71	-1.64	4.07
D	Pro236	Leu244	-1.61	3.90
D	Val132	Leu72	-1.53	4.02
D	Val234	Leu80	-1.51	3.97
D	Val132	Leu74	-1.44	4.35
D	Leu310	Thr137	-1.38	4.08
D	Pro135	Leu69	-1.32	4.15

D	Met330	Leu72	-1.25	3.90
D	Val136	Ser246	-1.07	4.24
D	Ala131	Leu69	-0.97	3.98
D	Arg235	Leu316	-0.82	3.94
D	Arg128	Val71	-0.81	4.25
D	Gln139	Thr247	-0.80	4.18
D	Val234	Pro125	-0.75	3.89
D	Lys311	Thr137	-0.72	3.98
D	Tyr138	Leu69	-0.70	4.24
D	Gly266	Phe245	-0.64	4.45
D	Ala131	Leu72	-0.63	4.18
D	Cys231	Lys78	-0.54	3.67
D	Pro135	Leu74	-0.52	4.27
D	Val234	Leu124	-0.50	4.26

Table S4. Protein-protein contacts between the D3R and βarr2 in D3R- βarr2-a-SK609 complex. **DIH**, **DH** and **D** denote ionic H-bond, H-bond and van der Waals interaction respectively.

Type	D3R	βarr2	Energy, Kcal/mol	Dist, Å
DIH	Arg128	Asp68	-28.57	3.30
DIH	Lys392	Glu135	-22.69	3.37
DH	Met134	Arg66	-6.03	3.89
DH	Ala131	Arg66	-5.59	3.78
D	Ile258	Leu155	-4.78	4.03
DH	Arg323	Leu72	-4.31	3.91
D	Thr308	Leu74	-2.36	3.94
DH	Gln144	Thr247	-2.19	3.88
D	Tyr255	Phe81	-1.97	4.02
D	Ser145	Phe245	-1.93	4.10
D	Ile258	Glu156	-1.77	3.99
D	Pro267	Phe76	-1.76	3.91
D	Pro135	Leu69	-1.74	4.18
D	Gln260	Ile82	-1.55	3.93
D	Tyr255	Ile82	-1.50	3.94
D	Pro267	Leu69	-1.26	4.03
DH	Arg254	Thr84	-1.23	4.07

D	Thr142	Tyr250	-1.22	3.86
D	Ile258	Phe150	-1.22	4.19
D	Pro135	Phe76	-1.20	3.68
D	Gln139	Arg66	-1.11	3.78
D	HSE137	Phe245	-1.05	4.17
D	Val132	Asp68	-0.98	4.12
D	Leu322	Leu72	-0.97	4.13
D	Gln139	Phe245	-0.96	4.23
D	Leu252	Leu155	-0.77	3.84
D	Val132	Leu72	-0.69	4.00
D	Gly268	Phe76	-0.68	4.12
D	Ala131	Asp68	-0.64	4.14
D	Ser257	Leu155	-0.61	4.15
D	Ser146	Phe245	-0.60	4.04
D	Leu322	Val71	-0.60	4.03
D	Ile258	Thr84	-0.59	4.16
D	Lys396	Glu135	-0.59	4.28
D	Val136	Phe245	-0.57	3.96
D	Glu389	Thr137	-0.54	3.94

Table S5. Protein-protein contacts between the D3R and βarr2 in D3R- βarr2-b-PRX complex. **DIH**, **DH** and **D** denote ionic H-bond, H-bond and van der Waals interaction respectively.

Type	D3R	βarr2	Energy, Kcal/mol	Dist, Å
DIH	Glu324	Lys78	-26.81	3.66
DIH	Lys253	Asp241	-25.98	3.26
DIH	Ser146	Lys139	-21.37	3.53
DIH	Ser146	Arg66	-14.26	3.68
DIH	Arg128	Asp70	-13.10	3.56
DH	Leu322	Lys78	-12.36	3.46
DH	Gln139	Arg286	-5.64	3.85
DH	Gln316	Leu80	-4.76	3.52
DH	Gln139	Cys141	-3.11	3.75
D	Ile124	Leu72	-3.04	3.94
DH	Asn65	Leu69	-2.98	3.79
D	Glu389	Leu69	-2.95	4.11
DH	Leu264	Asn312	-2.84	3.98
DH	Thr64	Asp70	-2.65	3.77
DH	Asn65	Val71	-2.56	3.66
DH	Asn387	Val71	-2.26	4.00
D	Met330	Leu72	-2.14	3.99
DH	Val213	Phe245	-1.86	3.66
I	Arg218	Glu314	-1.83	3.52
DH	Gly268	Ala311	-1.81	3.87
D	Arg128	Phe76	-1.80	3.94
D	Gln316	Phe81	-1.74	3.97
D	Val136	Tyr250	-1.72	3.99

DH	Val214	Phe245	-1.58	4.02
D	Leu264	Glu314	-1.58	4.07
D	Val136	Leu130	-1.50	3.94
D	Ala263	Glu314	-1.49	4.00
D	Thr62	Asp70	-1.44	3.98
D	Ala59	Leu69	-1.42	4.04
D	Val331	Leu72	-1.35	4.05
D	Tyr383	Val71	-1.26	4.06
D	Val320	Leu316	-1.23	3.49
D	Val132	Phe245	-1.22	4.07
D	Ala131	Phe76	-1.08	4.12
D	Leu252	Val307	-1.07	4.32
D	Phe390	Val71	-1.06	4.09
D	Ala131	Leu74	-1.05	3.67
D	Thr64	Val71	-0.92	4.37
D	Val136	Cys141	-0.91	4.10
D	Val214	Thr247	-0.88	4.26
D	Val214	Cys243	-0.87	4.01
DH	Gln139	Gln131	-0.86	3.93
D	Cys259	Thr247	-0.86	4.36
DH	Leu252	Arg323	-0.84	3.66
D	Val136	Ile242	-0.82	4.20
D	Leu252	Thr305	-0.82	4.11
D	Arg128	Leu72	-0.79	4.21
D	Val68	Val71	-0.75	4.18
D	Val334	Leu72	-0.73	3.97
D	Ile258	Gln249	-0.72	4.26
D	Pro135	Ile242	-0.64	4.27
D	Tyr212	Phe245	-0.64	4.09
D	Ile258	Thr247	-0.62	4.12
D	Met330	Val71	-0.61	4.15
D	Leu252	Leu319	-0.57	4.23
D	Glu324	Phe245	-0.54	4.07
D	Ala327	Leu72	-0.54	3.96
D	Ala327	Leu74	-0.53	4.23
D	Thr62	Asp68	-0.52	4.11
D	Ala131	Phe245	-0.52	4.16
D	Leu264	Ala311	-0.52	3.76

Table S6. Protein-protein contacts between the D3R and βarr2 in D3R-βarr2-b-SK609 complex. **DIH**, **DH** and **D** denote ionic H-bond, H-bond and van der Waals interaction respectively.

Type	D3R	βarr2	Energy, Kcal/mol	Dist, Å
DIH	Arg128	Asp70	-38.05	3.33
DIH	Arg218	Glu314	-34.58	3.32
DIH	Lys311	Glu157	-23.37	3.43
DIH	Lys311	Glu156	-19.80	3.46

DIH	Ser145	Lys139	-18.74	3.64
DIH	Ser146	Arg66	-15.00	3.69
DIH	Arg283	Glu156	-13.82	3.91
DH	Gln144	Lys139	-7.66	3.50
DH	Gln139	Cys141	-4.13	3.77
DH	Ala131	Tyr64	-3.61	3.37
DH	Asn387	Val71	-3.10	3.78
DH	Arg283	Ser154	-2.50	3.59
D	Arg128	Leu72	-2.37	4.08
D	Ile388	Lys158	-2.26	4.15
DH	Thr62	Glu67	-2.03	3.82
D	Arg128	Leu74	-1.86	4.00
D	Val214	Ser246	-1.70	3.99
D	Arg318	Phe118	-1.63	4.18
DH	Gln316	Leu80	-1.61	3.95
D	Ala327	Leu74	-1.55	3.83
D	Asn65	Val71	-1.41	3.89
D	Val132	Leu74	-1.35	4.21
D	Pro135	Phe62	-1.32	4.12
D	Ser257	Glu314	-1.19	4.02
D	Met330	Leu72	-1.18	4.05
D	Val132	Phe76	-1.16	4.02
D	Ala131	Phe76	-1.13	4.28
D	Ile124	Leu72	-1.05	3.73
D	Pro135	Leu244	-1.05	4.27
D	Phe390	Val71	-1.00	4.18
D	Leu60	Val71	-0.97	3.74
D	Ser285	Lys53	-0.93	3.76
DH	Gln139	Gln131	-0.84	4.01
D	Gln139	Arg286	-0.82	3.90
D	Val132	Phe245	-0.76	4.07
D	Pro135	Ile242	-0.75	3.99
DH	Gln139	Leu130	-0.71	3.94
D	Ser285	Phe55	-0.65	4.05
D	Glu324	Gly73	-0.65	4.09
D	Leu264	Asn312	-0.64	4.06
D	Pro321	Ile82	-0.64	3.81
D	Pro267	Asn312	-0.62	3.86
D	Tyr256	Glu314	-0.61	4.00
D	Gln316	Phe81	-0.61	4.13
D	Val136	Ile242	-0.60	4.20
D	Ile258	Thr247	-0.59	3.82
D	Met134	Leu244	-0.52	3.97

Table S7. Important protein-protein contacts in the final conformation of D3R-Gi-PRX complex. **DIH**, **DH** and **D** denote ionic H-bond, H-bond and van der Waals interaction respectively.

	D3R	Gα	Energy, kcal/mol	Dist, Å
DIH	Arg226	Asp337	-35.44	3.36
DIH	Arg222	Asp309	-30.21	3.24
DIH	Arg323	Glu318	-29.75	3.41
DIH	Arg305	Asp261	-28.60	3.22
DIH	Arg254	Asp341	-20.11	3.54
DIH	Arg323	Phe354	-18.01	3.78
DIH	Lys221	Glu297	-17.76	3.59
DIH	Arg318	Asp315	-14.44	3.60
DIH	Lys311	Asp261	-13.82	3.53
DIH	Arg222	Glu308	-13.55	3.45
DH	Gln227	Lys271	-6.66	3.86
DH	His140	Asn346	-4.90	3.85
DH	Asn228	Tyr296	-3.18	3.68
DH	Thr308	Glu318	-3.16	3.63
D	Arg254	Ile344	-2.12	3.93
D	Leu224	Ile303	-1.95	4.19
D	Ala327	Leu353	-1.62	3.94
D	Gln227	Phe323	-1.60	3.93
D	Val136	Thr340	-1.57	3.84
D	Thr308	Lys317	-1.57	4.08
D	Val136	Ile343	-1.52	4.30
D	Leu224	Leu268	-1.45	3.99
D	Leu224	Phe323	-1.43	3.92
D	Pro135	Ile343	-1.23	3.95
D	Pro317	Lys317	-1.12	4.14
D	Lys326	Leu353	-1.07	3.99
D	Val136	Phe336	-1.01	4.32

Table S8. Important protein-protein contacts in the final conformation of D3R-Gi-SK609 complex. **DIH**, **DH** and **D** denote ionic H-bond, H-bond and van der Waals interaction respectively.

	D3R	Gα	Energy, kcal/mol	Dist, Å
DIH	Arg254	Asp341	-36.77	3.39
DIH	Arg305	Asp261	-32.41	3.54
DIH	Arg220	Glu297	-30.96	3.24
DIH	Arg222	Asp309	-30.61	3.31

DIH	Arg226	Asp337	-30.27	3.24
DIH	Arg235	Glu276	-26.23	3.79
DIH	Arg278	Asp315	-25.18	3.66
DIH	Arg318	Asp261	-23.65	3.47
DIH	Arg323	Glu318	-18.18	3.39
DIH	Arg219	Glu308	-17.63	3.53
DIH	Arg222	Glu297	-15.97	3.65
DIH	Arg219	Asp309	-15.53	3.60
DIH	Lys277	Glu318	-15.00	3.46
DH	Asn228	Ala326	-4.88	3.66
DH	Tyr138	Asp350	-4.32	3.67
DH	Ser307	Phe354	-4.10	3.54
DH	Gln230	Glu275	-3.47	3.66
D	Gln227	Phe323	-2.52	4.07
D	Thr225	HSD322	-2.39	3.83
D	Phe386	Leu353	-2.33	4.09
D	Val320	Glu318	-2.22	4.06
D	Arg323	Phe354	-1.98	4.02
D	Leu224	Thr321	-1.95	3.77
D	Asn228	Cys325	-1.80	3.97
D	Ala327	Leu353	-1.73	3.98
D	Leu301	Thr316	-1.53	3.88
D	Lys326	Gly352	-1.48	3.62
D	Ile258	Leu348	-1.43	3.89
D	Tyr138	Ile343	-1.25	4.11
D	Leu301	Asp315	-1.17	4.19
D	Arg323	Leu353	-1.09	3.93
D	Arg128	Cys351	-0.44	4.00

Movie S1. The movie obtained from the principal component analysis (PCA) of the trajectory from the MD simulation of the D3R-PRX complex. The movie depicted the first principal component displacement of helices at the cytoplasmic side of D3R.

Movie S2. The movie obtained from the principal component analysis (PCA) of the trajectory from the MD simulation of the D3R-SK609 complex. The movie depicted the first principal component displacement of helices at the cytoplasmic side of D3R.
